# General solution of the chemical master equation and modality of marginal distributions for hierarchic first-order reaction networks

**DOI:** 10.1007/s00285-018-1205-2

**Published:** 2018-01-20

**Authors:** Matthias Reis, Justus A. Kromer, Edda Klipp

**Affiliations:** 1 Institute of Biology, Theoretical Biophysics, Humboldt-Universität zu Berlin, 10117 Berlin, Germany; 2 Center for Advancing Electronics Dresden, Technische Universität Dresden, 01062 Dresden, Germany

**Keywords:** Hierarchically linear ODE system, Hierarchic reaction network, Chemical master equation, Conditional multimodality, Compound process, Bursting process, Generalized poisson distribution, Generalized binomial distribution, Nuclear decay chain, Transcription–translation model, 92C45, 60J27, 05A15

## Abstract

Multimodality is a phenomenon which complicates the analysis of statistical data based exclusively on mean and variance. Here, we present criteria for multimodality in hierarchic first-order reaction networks, consisting of catalytic and splitting reactions. Those networks are characterized by independent and dependent subnetworks. First, we prove the general solvability of the Chemical Master Equation (CME) for this type of reaction network and thereby extend the class of solvable CME’s. Our general solution is analytical in the sense that it allows for a detailed analysis of its statistical properties. Given Poisson/deterministic initial conditions, we then prove the independent species to be Poisson/binomially distributed, while the dependent species exhibit generalized Poisson/Khatri Type B distributions. Generalized Poisson/Khatri Type B distributions are multimodal for an appropriate choice of parameters. We illustrate our criteria for multimodality by several basic models, as well as the well-known two-stage transcription–translation network and Bateman’s model from nuclear physics. For both examples, multimodality was previously not reported.

**Electronic supplementary material:**

The online version of this article (10.1007/s00285-018-1205-2) contains supplementary material, which is available to authorized users.

## Introduction

The development of single-molecule methods such as Fluorescence *in situ* Hybridization (FISH) has resulted in considerable advances in fundamental research in biology (Trcek et al. 2011, 2012). Those methods provide researchers with discrete, single-molecule data on (bio-)chemical reaction networks. The measured molecule numbers are often small, requiring a modeling approach that accounts for the discreteness of the molecule numbers (Klipp et al. 2009).

Networks of stochastically reacting molecule species are often described by the Chemical Master Equation (CME) (Gardiner 2009; Kampen 2011). The CME is a difference–differential equation. By using Gillespie’s Stochastic Simulation Algorithm (Gillespie 1977), an exact realization of the CME’s underlying Markov jump process is sampled and an indirect solution is obtained (Gillespie 1992). The CME may also be studied by approximation methods such as finite state projection (Munsky and Khammash 2006), or exactly using analytical approaches. Stationary solutions to the CME are known for a wide range of chemical reaction networks (Kampen 1976; Anderson et al. 2010). However, time-dependent, analytical solutions only exist for monomolecular reaction networks (Gans 1960; Jahnke and Huisinga 2007), consisting exclusively of conversion $S_{i}\leftrightarrow S_{j}$, degradation $S_{i}\rightarrow \emptyset$ and production $\emptyset \rightarrow S_{j}$ reactions, where $S_{i}$ defines a species of molecules. In contrast to numerical solution methods, analytical solutions allow for an abstract study of reaction networks, independently of the concrete value of reaction rate constants and network graphs.

Here, we present an extension of the class of analytically solvable CME’s to a subset of first-order reactions, namely hierarchic first-order networks. In addition to monomolecular reactions, first-order reactions include (auto-)catalytic and splitting reactions, and therefore allow for more real-world applications. As a subset of those networks, we define hierarchic networks (Def. 3), characterized by a division into independent and dependent subnetworks. Common examples of hierarchic networks are the transcription–translation model in molecular biology (Friedman et al. 2006; Shahrezaei and Swain 2008), or nuclear decay chains in physics (Bateman 1910; Pressyanov 2002), both of which we discuss here.

In contrast to monomolecular networks, first-order networks result in marginal distributions of individual species that are not unimodal in general, as we show in Theorems 3 and 4. These distributions are poorly characterized by mean and variance, two essential measures used to describe statistical data in natural sciences. On one hand, characterizing multimodal distributions by mean and variance is unintuitive, since the mean is often a very unlikely outcome. On the other hand, many unimodal distributions, such as the Gaussian distribution, are fully specified by the first two cumulants, i.e. mean and variance, or just the mean in case of the Poisson distribution. Contrastingly, an infinite number of cumulants can be necessary to fully specify the multimodal distributions derived in this article. We therefore argue that CME approximation methods based on the first two cumulants or moments, such as the moment-closure method (Milner et al. 2012), yield insufficient results when reaction networks lead multimodal molecule distributions. Albeit to a lesser degree, this hold true even when more than the first two moments are used to approximate the CME’s solution in approaches such as Constantino et al. (2016). Such distributions have already been reported for networks that include two-state or three-state random variables such as promoter switches studied in the context of gene-regulation (Shahrezaei and Swain 2008; Thomas et al. 2014). In the same context, a maximum-entropy approach has been used to approximate multimodal molecule distributions successfully using the first seven moments (Andreychenko et al. 2017).

In the present paper, we develop an exact theoretical description for hierarchic first-order networks, based on probability generating functions. Hierarchic first-order networks are introduced in Sect. 2, followed by the main results in Sect. 3. In Sect. 3.1, we establish the existence of analytical solutions (Theorem 1) and derive the *joint* probability generating functions (Proposition 2), which are complete characterizations of the underlying statistics. Then, the analytical form of the *marginal *distributions of individual species is derived in Sect. 3.2. We show that the independent part of the network exhibits Poissonian and Binomial marginal distributions, while the dependent part is described by Discrete Compound Poisson (DCP) and Kathri Type B (KTB) marginal distributions. Next, we present criteria for the marginal distributions to be multimodal in Sect. 3.3. Since Poissonian/Binomial distributions are generally unimodal, the independent part exhibits unimodal marginal distributions (Theorem 2). In contrast, DCP/KTB marginal distributions from the dependent part can lead to multimodality under quite general conditions (Theorems 3 and 4). We illustrate these general results by several basic models (Sect. 4.1) and two real-world models (Sect. 4.2). Among these are the transcription–translation model (Example 5) and Bateman’s model for nuclear decay chains (Example 6). For the former, multimodality was previously only discussed in an extended three-stage model variant (Shahrezaei and Swain 2008), whereas Theorem 3 proves the protein numbers to be multimodally distributed even in the simple two-species version. To the best of our knowledge, our exact solution to the CME for Bateman’s model of nuclear decay chains is a novel result, applying the techniques developed in the preceding sections. We apply Theorem 4 to show multimodality for Bateman’s model.

The derivation of probability mass functions from generating functions is reviewed in Appendix A.1.

## Introduction to hierarchic first-order networks

### Chemical master equation and characteristic ODEs

First of all, we define a network of *n* species with *m* chemical reactions by the $n\times m$ stoichiometric matrices ${\mathbf {Q}}=(Q_{ij})$ and ${\mathbf {R}}=(R_{ij})$, where $Q_{ij}$, $R_{ij}\in {\mathbb {N}}_{0}$. These matrices enable us to write

$$\begin{aligned} {\mathbf {Q}}^{\text {T}}{\mathbf {S}}{\mathop {\rightarrow }\limits ^{{\mathbf {k}}}}{\mathbf {R}}^{\text {T}}{\mathbf {S}}, \end{aligned}$$

with ${\mathbf {S}}=(S_{1},\ldots ,S_{n})^{\text {T}}$ being the vector of species and ${\mathbf {k}}=(k_{1},\ldots ,k_{m})^{\text {T}}$ the vector of rate constants.

In the present paper, we consider discrete and stochastic models of biochemical reaction networks. Let $P({\mathbf {x}},t|{\mathbf {\mathbf {x}}}_{0},t_{0})$ be the probability distribution for the number of molecules ${\mathbf {x}}$ of each species at time *t*, given the initial condition ${\mathbf {x}}_{0}$ at $t_{0}$. This distribution is described by the Chemical Master Equation, a difference–differential equation defined by^1^

1 $$\begin{aligned} \partial _{t}P({\mathbf {x}},t)=&\sum _{i=1}^{m}\Big (\alpha _{i}({\mathbf {x}}-{\mathbf {n}}_{i})P({\mathbf {x}}-{\mathbf {n}}_{i},t)-\alpha _{i}({\mathbf {x}})P({\mathbf {x}},t)\Big ), \end{aligned}$$

where

2 $$\begin{aligned} \alpha _{i}({\mathbf {x}}):=&k_{i}\prod _{j=1}^{n}\frac{x_{j}!}{(x_{j}-Q_{ji})!}\ \text {and}\ {\mathbf {n}}_{i}:=\text {col}_{i}({\mathbf {R}}-{\mathbf {Q}}). \end{aligned}$$

In the last equation $\text {col}_{i}$ denotes the *i*th column of a matrix. The CME (1) is based on the Markovian assumption and is derived in detail in Gardiner (2009) or Kampen (2011). Applicable for (multivariate) distributions of discrete random variables, we introduce the concept of *generating functions*:

#### Definition 1

(*Generating function*) A *probability generating function* (PGF) is a power series^2^

3 $$\begin{aligned} g({\mathbf {s}},t):=\sum _{{\mathbf {x}}={\mathbf {0}}}^{\infty }{\mathbf {s}}^{{\mathbf {x}}}P({\mathbf {x}},t) \end{aligned}$$

defined for ${\mathbf {s}}\in {\mathbb {C}}^{n}$ within some radius of convergence $\le 1$ in each coordinate respectively.

In the univariate case, a large list of correspondences between PGFs and distributions is provided by Johnson et al. (2005). Next, we represent the Master Eq. (1) as a partial differential equation (PDE) in terms of a generating function:

#### Lemma 1

The Master Eq. (1) can equivalently be expressed as the partial differential equation

4 $$\begin{aligned} \partial _{t}g({\mathbf {s}},t)&=\sum _{i=1}^{m}k_{i}\left( \prod _{j=1}^{n}s_{j}^{R_{ji}}-\prod _{j=1}^{n}s_{j}^{Q_{ji}}\right) \left( \prod _{j=1}^{n}\partial _{s_{j}}^{Q_{ji}}\right) g({\mathbf {s}},t). \end{aligned}$$

This result can be found in textbooks such as Gardiner (2009), Sect. 11.6.4, p. 297. We provide a proof in “Appendix A.2” to the convenience of the reader.

We define *first-order networks* as those obeying a first-order PDE for the generating function ($\sum _{j=1}^{n}Q_{ji}\le 1$), or equivalently a linear ordinary differential equation (ODE) system for the moments. In terms of chemical reactions, first-order networks consist exclusively of reactions of the type $S_{j}\rightarrow \sum _{l}R_{li}S_{l}$, i.e. monomolecular plus splitting and (auto-)catalytic reactions. First-order PDEs are solvable by the method of characteristics,^3^ a method to convert the PDE solution problem into ODEs. The basic idea is that the solution of the PDE is given by a set of integral curves ${\mathbf {s}}(\tau )$ called *characteristic curves*, parametrized by some $\tau \in {\mathbb {R}}$. We obtain the ODE system, describing these curves ${\mathbf {s}}(\tau )$, by interpreting (4) as a total derivative, i.e.

5 $$\begin{aligned} \frac{dg({\mathbf {s}}(\tau ),t(\tau ))}{d\tau }=\frac{\partial g({\mathbf {s}}(\tau ),\tau )}{\partial t}\frac{dt}{d\tau }+\sum _{i=1}^{n}\frac{\partial g({\mathbf {s}}(\tau ),\tau )}{\partial s_{i}}\frac{ds_{i}(\tau )}{d\tau }{\mathop {=}\limits ^{!}}&f({\mathbf {s}}(\tau ),\tau )g({\mathbf {s}}(\tau ),\tau ). \end{aligned}$$

Then, the *characteristic system of ODEs*, obtained by comparing the coefficients of (5) and (4), reads

6 $$\begin{aligned} \frac{dt}{d\tau }&=1\nonumber \\ \frac{ds_{{j}}(\tau )}{d\tau }&=-\sum _{i=1}^{m}k_{i}{Q_{ji}}\left( \prod _{{l}=1}^{n}\big (s_{{l}}(\tau )\big )^{R_{{l}i}}-{s_{j}(\tau )}\right) \qquad {(j\in \{1,\ldots ,n\})} \end{aligned}$$

7 $$\begin{aligned} \frac{dg({\mathbf {s}}(\tau ),t(\tau ))}{d\tau }&=\underbrace{\sum _{i=1}^{m}\sum _{j=1}^n k_{i}{(1-{Q_{ji}})}\left( \prod _{l=1}^{n}\big (s_{l}(\tau )\big )^{R_{li}}-1\right) }_{:=f({\mathbf {s}}(\tau ),\tau )}g({\mathbf {s}}(\tau ),\tau ). \end{aligned}$$

In case we not only require $\sum _{j=1}^{n}Q_{ji}\le 1$, but also $\sum _{j=1}^{n}R_{ji}\le 1$, the equations for $\frac{ds_{i}(\tau )}{d\tau }$ and $\frac{dg({\mathbf {s}}(\tau ),t(\tau ))}{d\tau }$ are linear and we are restricted to *monomolecular reactions*. In consequence, the characteristic ODE system is solvable and we obtain a general solution of the CME, as shown in Gans (1960).^4^ For the complete definition of the ODE system, we specify the initial conditions

8 $$\begin{aligned} {\mathbf {s}}^{0}:&={\mathbf {s}}(0)\nonumber \\ g({\mathbf {s}}^{0},0)&=d({\mathbf {s}}^{0}). \end{aligned}$$

The generating function $d({\mathbf {s}}^{0})$ specifies the initial distribution. Here, we study *deterministic* initial conditions $d({\mathbf {s}}^{0})=\big ({\mathbf {s}}^{0}\big )^{{\mathbf {x}}_{0}}$ and *product Poissonian* initial conditions

$$\begin{aligned} d({\mathbf {s}}^{0})=\exp \left[ \langle {\mathbf {x}}\rangle _{0}\cdot ({\mathbf {s}}^{0}-{\mathbf {1}})\right] , \end{aligned}$$

where $\big <{\mathbf {x}}\big >_{0}$ is the expected number of molecules at $t=0$.

### Hierarchically linear ODE systems and reaction networks

Compared to monomolecular reaction networks, a more general subset of first-order networks are hierarchically linear networks, which we characterize in this article.

#### Definition 2

(*Hierarchically linear ODE system*) Let ${\mathbf {x}}_{i}(t)$, ${\mathbf {f}}_{i}(\cdot ,t)$ and ${\mathbf {h}}_{i}(\cdot ,t)$ be continuously differentiable, vector- and matrix-valued functions respectively.^5^ An ODE system exhibiting the structure

9 $$\begin{aligned} \frac{d{\mathbf {x}}_{1}}{dt}&={\mathbf {f}}_{1}({\mathbf {x}}_{2},{\mathbf {x}}_{3},\ldots ,{\mathbf {x}}_{n})+{\mathbf {h}}_{1}({\mathbf {x}}_{2},{\mathbf {x}}_{3},\ldots ,{\mathbf {x}}_{n}){\mathbf {x}}_{1}\nonumber \\ \frac{d{\mathbf {x}}_{2}}{dt}&={\mathbf {f}}_{2}({\mathbf {x}}_{3},{\mathbf {x}}_{4},\ldots ,{\mathbf {x}}_{n})+{\mathbf {h}}_{2}({\mathbf {x}}_{3},{\mathbf {x}}_{4},\ldots ,{\mathbf {x}}_{n}){\mathbf {x}}_{2}\nonumber \\&\vdots \nonumber \\ \frac{d{\mathbf {x}}_{n}}{dt}&={\mathbf {f}}_{n}+{\mathbf {h}}_{n}{\mathbf {x}}_{n} \end{aligned}$$

is called a *hierarchically linear ODE system*.

The next proposition gives a reason for our interest in hierarchically linear ODE systems:

#### Proposition 1

All hierarchically linear ODE systems are solvable by means of matrix exponentials.

#### Proof

We inspect a system of two levels only, since an extension to the ${\mathbf {n}}$-dimensional case follows by mathematical induction. Then, the system for $n=2$

$$\begin{aligned} {\dot{\mathbf {x}}}_{1}&={\mathbf {f}}_{1}({\mathbf {x}}_{2})+{\mathbf {h}}_{1}({\mathbf {x}}_{2}){\mathbf {x}}_{1}\\ {\dot{\mathbf {x}}}_{2}&={\mathbf {f}}_{2}+{\mathbf {h}}_{2}{\mathbf {x}}_{2} \end{aligned}$$

has a solution in terms of simple matrix exponentials if ${\mathbf {h}}_{1}({\mathbf {x}}_{2}(t_{1}))$ and ${\mathbf {h}}_{1}({\mathbf {x}}_{2}(t_{2}))$ commute, i.e.

$$\begin{aligned} {\mathbf {h}}_{1}({\mathbf {x}}_{2}(t_{1})){\mathbf {h}}_{1}({\mathbf {x}}_{2}(t_{2}))={\mathbf {h}}_{1}({\mathbf {x}}_{2}(t_{2})){\mathbf {h}}_{1}({\mathbf {x}}_{2}(t_{1}))~. \end{aligned}$$

In this case, the solution can be written^6^ as

10 $$\begin{aligned} {\mathbf {x}}_{1}(t)&=\exp \left\{ \int _{0}^{t}{\mathbf {h}}_{1}({\mathbf {x}}_{2}(t'))dt'\right\} {\mathbf {x}}_{1}^{0}\nonumber \\&\quad +\exp \left\{ \int _{0}^{t}{\mathbf {h}}_{1}({\mathbf {x}}_{2}(t'))dt'\right\} \int _{0}^{t}\exp \left\{ -\int _{0}^{t'}{\mathbf {h}}_{1}({\mathbf {x}}_{2}(t''))dt''\right\} {\mathbf {f}}_{1}({\mathbf {x}}_{2}(t'))dt' \end{aligned}$$

11 $$\begin{aligned} {\mathbf {x}}_{2}(t)&=\exp \{{\mathbf {h}}_{2}t\}{\mathbf {x}}_{2}^{0}+\exp \{{\mathbf {h}}_{2}t\}\int _{0}^{t}\exp \{-{\mathbf {h}}_{2}t'\}{\mathbf {f}}_{2}dt'~. \end{aligned}$$

If the matrix-valued function ${\mathbf {h_{1}}}$ does not commute for two different instants of time, the matrix exponent is expressed in terms of Magnus series (see Magnus 1954). Even in that case $[{\mathbf {h}}_{1}({\mathbf {x}}_{2}(t_{1})),{\mathbf {h}}_{1}({\mathbf {x}}_{2}(t_{2}))]\not =\varvec{0}$, it is still possible to write down an analytical expression in terms of matrix exponentials. $\square$

A hierarchically linear ODE system translates to the study of chemical reaction networks modeled by the CME as follows:

#### Definition 3

(*Hierarchic first-order reaction network*) A first-order reaction network, described by a hierarchically linear characteristic ODE system is called hierarchic first-order reaction network.

### Two-level hierarchic networks

In contrast to the general form of the preceding definition, we focus on *two-level* hierarchic networks, composed of two subsystems. That is, we have $n_{\text {ind}}$ independent species ${\mathbf {S}}_{\text {ind}}=(S_{1},\ldots ,S_{n_{\text {ind}}},0,\ldots ,0)^{\text {T}}$ and $n_{\text {dep}}$ dependent species ${\mathbf {S}}_{\text {dep}}=(0,\ldots ,0,S_{n_{\text {ind}}+1},\ldots ,S_{n})^{\text {T}}$, where $n_{\text {ind}}+n_{\text {dep}}=n$. We express a two-level hierarchic network in terms of chemical reactions as

12 $$\begin{aligned} {\mathbf {Q}}^{\text {T}}{\mathbf {S}}_{\text {ind}}{\mathop {\rightarrow }\limits ^{{\mathbf {k}}}}&{\mathbf {R}}^{\text {T}}{\mathbf {S}}_{\text {ind}}+{\mathbf {R}}^{\text {T}}{\mathbf {S}}_{\text {dep}}\qquad \text {(first order reactions, system I)}, \end{aligned}$$

13 $$\begin{aligned} {\mathbf {Q}}^{\text {T}}{\mathbf {S}}_{\text {dep}}{\mathop {\rightarrow }\limits ^{{\mathbf {k}}}}&{\mathbf {R}}^{\text {T}}{\mathbf {S}}_{\text {dep}}\qquad \text {(monomolecular reactions, system~II)}. \end{aligned}$$

The first-order reactions’ products are split into two parts: ${\mathbf {S}}_{\text {ind}}$ appears only once per reaction ($\sum _{j=1}^{n_{\text {ind}}}R_{ji}\le 1$ for $i\in \{1,\ldots ,m\}$), while an arbitrary number of molecules ${\mathbf {S}}_{\text {dep}}$ is allowed. The monomolecular reactions are defined by $\sum _{j=n_{\text {ind}}+1}^{n}R_{ji}\le 1$ for $i\in \{1,\ldots ,m\}$.

**Fig. 1:**
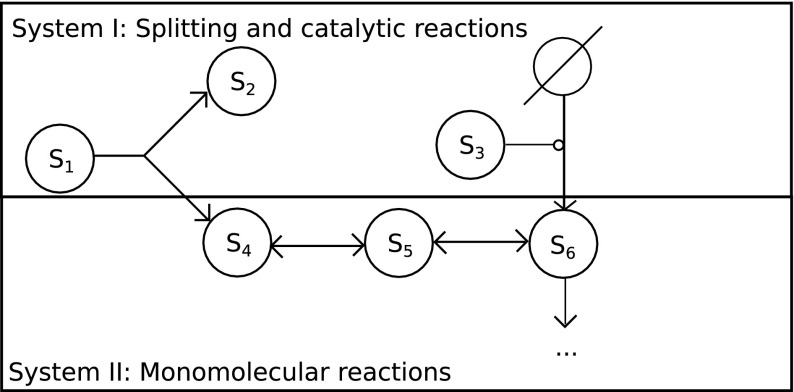
Scheme depicting a hierarchic first-order network. In system I, all non-monomolecular reactions take place, such as splitting or catalytic reactions, while in system II only monomolecular reactions take place

Note that by ignoring ${\mathbf {S}}_{\text {dep}}$, we might study ${\mathbf {Q}}^{\text {T}}{\mathbf {S}}_{\text {ind}}\rightarrow {\mathbf {R}}^{\text {T}}{\mathbf {S}}_{\text {ind}}$ *independently *of the monomolecular reactions (13), and obtain another monomolecular network, since $\sum _{j=1}^{n_{\text {ind}}}R_{ji}\le 1$ for $i\in \{1,\ldots ,m\}$. We therefore refer to (12) as *independent* part and (13) as *dependent* part of the hierarchic system, since the latter cannot be modeled independently of system I. Figure 1 depicts an example of such a hierarchic first-order two-level system.

Mathematically, (12) and (13) translate to a hierarchically linear characteristic ODE system. That is, the general form (6) is constrained to

14 $$\begin{aligned} \frac{ds_{j}}{dt}&=-\sum _{i=1}^{m}Q_{ji}k_{i}\left\{ \left[ \prod _{l=n_{\text {ind}}+1}^{n}s_{l}{}^{R_{li}}\right] \left( \sum _{l=1}^{n_{\text {ind}}}R_{li}s_{l}+\delta _{\sum _{l=1}^{n_{\text {ind}}}R_{li},0}\right) -Q_{ji}s_{j}\right\} \nonumber \\&\quad (j\in \{1,\ldots n_{\text {ind}}\}), \end{aligned}$$

15 $$\begin{aligned} \frac{ds_{j}}{dt}&=-\sum _{i=1}^{m}Q_{ji}k_{i}\left( \sum _{l=n_{\text {ind}}+1}^{n}R_{li}s_{l}+\delta _{\sum _{l=n_{\text {ind}}+1}^{n}R_{li},0}-Q_{ji}s_{j}\right) \nonumber \\&\quad (j\in \{n_{\text {ind}}+1,\ldots n\}). \end{aligned}$$

Note that the algebraic hierarchy is inverse to the reaction network hierarchy. The dependent part of the reaction network (system II) is described by an autonomous ODE system, while the independent part (system I) is non-autonomous due to the term $\prod _{l=n_{\text {ind}}+1}^{n}s_{l}{}^{R_{li}}$.

The monomolecular system II can be expressed more compactly as

$$\begin{aligned} S_{j}&{\mathop {\rightarrow }\limits ^{\alpha _{jk}}}S_{k}\\ S_{j}&{\mathop {\rightarrow }\limits ^{\alpha _{j0}}}\emptyset , \end{aligned}$$

where $j,k\in \{n_{\text {ind}}+1,\ldots ,n\}$ and ${\mathbf {A}}:=(\alpha _{ij})$ is the matrix holding the conversion rates. Using that matrix, we rewrite (15) as

16 $$\begin{aligned} \frac{d{\mathbf {s}}_{\text {dep}}}{dt}=-{\mathbf {A}}^{\text {T}}({\mathbf {s}}_{\text {dep}}-{\mathbf {1}}). \end{aligned}$$

The inversion of the hierarchic and algebraic structure, mentioned in the previous paragraph, also becomes clear by the traditional rate equations for the concentration vector ${\mathbf {C}}_{\text {dep}}$ of system II

17 $$\begin{aligned} \frac{d{\mathbf {C}}_{\text {dep}}}{dt}={\mathbf {A}}{\mathbf {C}}_{\text {dep}}+\text {influx from system I}. \end{aligned}$$

It was shown by Jahnke and Huisinga (2007) using a statistical argument that the solution to the monomolecular CME is a product Poisson distribution, given Poissonian initial conditions, and a multinomial distribution, given deterministic initial conditions. Thereby, the parameters of the distributions are given by the traditional rate Eq. (17). This parametrization is also evident by the characteristic ODEs (16) because these can be expressed using the transposed Jacobian matrix of (17).

However, it also becomes clear that the exact solution to the CME for a general first-order network, is not fully parametrized by the traditional rate equations: The ODE system (13) is structurally different from the traditional rate equations for system I due to the time-dependent coefficients $\prod _{l=n_{\text {ind}}+1}^{n}s_{l}{}^{R_{li}}$. In contrast to the characteristic Eq. (14), the traditional rate equation system is generally autonomous if the rate constants $k_{i}$ are time-independent as we assume.

In order to highlight the relationship between traditional rate equations and characteristic ODEs for system II, we use the convention

18 $$\begin{aligned} {\mathbf {Q}}^{\text {T}}{\mathbf {S}}_{\text {ind}}&{\mathop {\rightarrow }\limits ^{{\mathbf {k}}}}{\mathbf {R}}^{\text {T}}{\mathbf {S}}_{\text {ind}}+{\mathbf {R}}^{\text {T}}{\mathbf {S}}_{\text {dep}}\ ,\nonumber \\ S_{j}&{\mathop {\rightarrow }\limits ^{\alpha _{jk}}}S_{k},\nonumber \\ S_{j}&{\mathop {\rightarrow }\limits ^{\alpha _{j0}}}\emptyset ,\nonumber \\ \emptyset&{\mathop {\rightarrow }\limits ^{{\mathbf {b}}}}{\mathbf {S}}, \end{aligned}$$

instead of (12) and (13) to fully express the two-level hierarchic reaction network. Note that we summarized influx reactions to both system I and II in (18), which yields the characteristic equations

$$\begin{aligned} \frac{ds_{j}}{dt}&=-\sum _{i=1}^{m}Q_{ji}k_{i}\left\{ \left[ \prod _{l=n_{\text {ind}}+1}^{n}s_{l}{}^{R_{li}}\right] \left( \sum _{l=1}^{n_{\text {ind}}}R_{li}s_{l}+\delta _{\sum _{l=1}^{n_{\text {ind}}}R_{li},0}\right) -Q_{ji}s_{j}\right\} \\&\qquad (j\in \{1,\ldots n_{\text {ind}}\})\\ \frac{d{\mathbf {s}}_{\text {dep}}}{dt}&=-{\mathbf {A}}^{\text {T}}({\mathbf {s}}_{\text {dep}}-{\mathbf {1}})\\ \frac{dg({\mathbf {s}},t)}{dt}&={\mathbf {b}}\cdot ({\mathbf {s}}-{\mathbf {1}})g({\mathbf {s}},t) \end{aligned}$$

instead of (14) and (15).

## Main results

### Solvability of hierarchic first-order networks

First, we show that the CME is solvable for any hierarchically linear reaction network.

#### Theorem 1

(Existence of analytical solutions for hierarchic first-order networks) The Master equation corresponding to a hierarchic first-order reaction network is analytically solvable.

#### Proof

Applying Proposition 1, we find that the characteristic system (6) is solvable as a linear equation system as ${\mathbf {s}}=e^{{\mathbf {C}}}{\mathbf {s}}^{0}+{\mathbf {v}}$, where ${\mathbf {C}}$ is a square matrix and ${\mathbf {v}}$ a column vector. For the initial condition of (7), we need ${\mathbf {s}}^{0}$. Since $e^{{\mathbf {C}}}$ is generally invertible, the characteristic system ${\mathbf {s}}=e^{{\mathbf {C}}}{\mathbf {s}}^{0}+{\mathbf {v}}$ can be solved for ${\mathbf {s}}^{0}$. This in turn yields the generating function and the distribution, defined as the coefficients of the former. $\square$

#### Remark 1

The term “analytical solution”, which is often used in a more intuitive sense, requires some explanation. One may argue that the Master equation is a system of linear ODEs and can always be formally solved by a matrix exponential as such. This remains true even for bimolecular networks such as the Lotka–Volterra model (Lotka 1925). However, a formal solution of the Master equation does not tell us anything about the structure of the distribution, if the matrix exponential cannot be calculated explicitly. Contrary to this non-analytical case, the generating function stated in the next proposition earns the word “analytical”, even if it also contains a matrix exponential. As shown in Sect. 3.2, the matrix exponential from the next proposition enables us to analyze properties of the distribution and may be used to find the stationary distribution, to compute moments and cumulants, etc.

#### Proposition 2

Let ${\mathbf {b}}={\mathbf {0}}$, i.e. no reactions^7^ $\emptyset {\mathop {\rightarrow }\limits ^{{\mathbf {b}}}}{\mathbf {S}}$. Given that the molecules are initially product Poisson distributed, i.e. $g({\mathbf {s}},0)=\exp \big [\big <{\mathbf {x}}\big >_{0}\cdot ({\mathbf {s}}-{\mathbf {1}})\big ]$, the generating function is given by

19 $$\begin{aligned} g({\mathbf {s}},t)&=\exp \Big \{\langle {\mathbf {x_{1}}}\rangle _{0}\cdot \big [e^{-\int _{0}^{t}{\mathbf {J}}({\mathbf {s}}_{\text {dep}},t',t)dt'}{\mathbf {s}}_{\text {ind}}-\int _{0}^{t}e^{-\int _{0}^{t'}{\mathbf {J}}({\mathbf {s}}_{\text {dep}},t'',t)dt''}{\mathbf {f}}({\mathbf {s}}_{\text {dep}},t',t)dt'-{\mathbf {1}}\big ]\nonumber \\&\qquad +\exp ({\mathbf {A}}t)\langle {\mathbf {x_{2}}}\rangle _{0}\cdot ({\mathbf {s}}_{\text {dep}}-{\mathbf {1}})\Big \}. \end{aligned}$$

In the last expression, the Jacobian ${\mathbf {J}}$ is defined by

20 $$\begin{aligned} J_{pq}({\mathbf {s}}_{\text {dep}},t',t)&:=\sum _{i=1}^{m}Q_{pi}k_{i}\left( 1-c_{i}({\mathbf {s}}_{\text {dep}},t',t)R_{qi}\right) \ ,\quad p,q\in \{1,\ldots ,n_{\text {ind}}\} \end{aligned}$$

with^8^ $[{\mathbf {J}}({\mathbf {s}}_{\text {dep}},t_{1},t),{\mathbf {J}}({\mathbf {s}}_{\text {dep}},t_{2},t)]={\mathbf {0}}$ and ${\mathbf {f}}$ is given by

21 $$\begin{aligned} f_{j}({\mathbf {s}}_{\text {dep}},t',t):=-\sum _{i=1}^{m}Q_{ji}k_{i}c_{i}({\mathbf {s}}_{\text {dep}},t',t)~,&\quad j\in \{1,\ldots ,n_{\text {ind}}\} \end{aligned}$$

where

22 $$\begin{aligned} c_{i}({\mathbf {s}}_{\text {dep}},t',t):=\prod _{l=n_{\text {ind}}+1}^{n}\Big (\exp \big \{-{\mathbf {A}}(t'-t)\big \}\epsilon _{l}\cdot ({\mathbf {s}}_{\text {dep}}-{\mathbf {1}})+1\Big )^{R_{li}}. \end{aligned}$$

For deterministic initial conditions, i.e. $P({\mathbf {x}},0)=\delta _{{\mathbf {x}},{\mathbf {x}}^{0}}$, where ${\mathbf {x}}^{0}=(x_{1}^{0},\ldots ,x_{n}^{0})^{\text {T}},$ we obtain

23 $$\begin{aligned} g({\mathbf {s}},t)&=\Big \{\prod _{i=1}^{n}\Big (\big [e^{-\int _{0}^{t}{\mathbf {J}}({\mathbf {s}}_{\text {dep}},t',t)dt'}{\mathbf {s}}_{\text {ind}}-\int _{0}^{t}e^{-\int _{0}^{t'}{\mathbf {J}}({\mathbf {s}}_{\text {dep}},t'',t)dt''}{\mathbf {f}}({\mathbf {s}}_{\text {dep}},t',t)dt'\big ]\epsilon _{i}^{(1)}\nonumber \\&\qquad +\exp ({\mathbf {A}}t)\epsilon _{i}^{(2)}\cdot ({\mathbf {s}}_{\text {dep}}-{\mathbf {1}})+1\Big )^{x_{i}^{0}}\Big \}~, \end{aligned}$$

with $$\varepsilon _{i}^{(1)}:=\text {col}_{i}{\mathbf {I}}_{n_{\text {ind}}\times n_{\text {ind}}}$$ and $$\varepsilon _{i}^{(2)}:=\text {col}_{i}{\mathbf {I}}_{n_{\text {dep}}\times n_{\text {dep}}}$$.

We postpone the proof to “Appendix A.3” and interpret this result first. Remember that the deterministic or product Poisson distributions, assumed as an initial distributions in the preceding proposition, imply that all random variables are uncorrelated for a fixed time *t*. This can be shown by using the cumulant generating function $\kappa (\xi )=\log (g(e^{{\mathbf {\xi }}}))$, that is given for a product Poisson distribution by

$$\begin{aligned} \kappa (\xi )=\log (e^{\big<x\big>_{1}(e^{\xi _{1}}-1)+\cdots +\big<x\big>_{n}(e^{\xi _{n}}-1)})&=\big<x\big>_{1}(e^{\xi _{1}}-1)+\cdots +\big <x\big >_{n}(e^{\xi _{n}}-1)~. \end{aligned}$$

Here, “uncorrelated” means that the covariances and higher order mixed cumulants, defined as the coefficients of $\kappa (\xi )$, are zero:

$$\begin{aligned} \partial _{\xi _{1},\ldots ,\xi _{n}}\Big (\big<x\big>_{1}(e^{\xi _{1}}-1)+\cdots +\big <x\big >_{n}(e^{\xi _{n}}-1)\Big )\Big |_{\xi ={\mathbf {0}}}=0. \end{aligned}$$

For monomolecular reaction networks, the variables remain uncorrelated after some time $t>0$, since a monomolecular system given Poissonian initial conditions stays Poissonian, as shown in Jahnke and Huisinga (2007), Proposition 2. Contrastingly, for first-order processes, covariances between the variables appear for $t>0$, because the exponent of Eq. (19) is in general not a first-order polynomial. In other words, monomolecular systems stay uncorrelated for uncorrelated initial conditions, while first-order systems do not.

We understand the correlations in terms of chemical reactions by the difference between the first-order splitting reaction $S_{j}\rightarrow S_{k}+S_{l}$ and the two monomolecular reactions $S_{j}\rightarrow S_{k}$ and $S_{j}\rightarrow S_{l}$: The molecules $S_{k}$ and $S_{l}$ appear simultaneously in the splitting reaction, while the two monomolecular conversions are statistically independent events.

### Analytical form of marginal distributions

As we show in the following, the hierarchic network structure yields marginal distributions that are best interpreted as generalized distributions. We follow Johnson et al. (2005) for the next definition.

#### Definition 4

(*Generalizing distribution*) The distribution ${\mathscr {P}}_{1}$ is *generalized* by the *generalizing distribution* ${\mathscr {P}}_{2}$:

$$\begin{aligned} {\mathscr {P}}\sim {\mathscr {P}}_{1}\bigvee {\mathscr {P}}_{2}&:\Leftrightarrow&g(s)=g_{1}(g_{2}(s)), \end{aligned}$$

where $g_{1}(s)$ and $g_{2}(s)$ are the corresponding generating functions.

Generalized distributions are related to mixing distributions by Gurland’s theorem (Gurland 1957), if certain requirements are met. Next, we define two classes of generalized distributions that arise as marginal distributions for hierarchic networks.

#### Definition 5

(*Discrete compound Poisson distribution (DCP)*) A discrete compound Poisson distribution ${\mathscr {P}}_{\text {DCP}}$ is a univariate distribution (Zhang et al. 2014), generalizing the Poisson distribution with another distribution ${\mathscr {P}}_{2}$:

$$\begin{aligned} {\mathscr {P}}_{\text {DCP}}\sim {\mathscr {P}}_{\text {Poisson}}\bigvee {\mathscr {P}}_{2}. \end{aligned}$$

The generating function is defined accordingly:

$$\begin{aligned} g_{\text {DCP}}(s):=g_{\text {Poisson}}(g_{2}(s))=e^{\lambda (g_{2}(s)-1)}=e^{\sum _{i=1}^{\infty }\lambda \alpha _{i}(s^{i}-1)}\ , \end{aligned}$$

where $\lambda >0$ and $\alpha _{i}\in [0,1]$ with $\sum _{i=1}^{\infty }\alpha _{i}=1$ are *parameters*. We introduce the notation

24 $$\begin{aligned} \text {DCP}^{N}:\Leftrightarrow \deg _{s}\sum _{i=1}^{\infty }\lambda \alpha _{i}(s^{i}-1)=N . \end{aligned}$$

The next type of distribution was previously defined by Khatri and Patel (1961) and will be important for our study of reaction networks given deterministic initial conditions:

#### Definition 6

(*Khatri’s Type B distribution*) Khatri’s Type B distribution ${\mathscr {P}}_{\text {KTB}}$ is a univariate distribution, generalizing the deterministic distribution with another distribution ${\mathscr {P}}_{2}$:

$$\begin{aligned} {\mathscr {P}}_{\text {KTB}}\sim {\mathscr {P}}_{\text {Deterministic}}\bigvee {\mathscr {P}}_{2}. \end{aligned}$$

The generating function is defined accordingly:

$$\begin{aligned} g_{\text {KTB}}(s):=\big (g_{2}(s)\big )^{\nu }=\Bigg (\sum _{i=0}^{\infty }\alpha _{i}s^{i}\Bigg )^{\nu }\ , \end{aligned}$$

$\alpha _{i}\in [0,1]$ are *parameters* and $\nu \in {\mathbb {N}}_{0}$ is the number of trials. Furthermore, $g_{2}(s)$ must be a generating function, i.e. $\sum _{i=1}^{\infty }\alpha _{i}=1$. We introduce the notation

25 $$\begin{aligned} \text {KTB}^{N}:\Leftrightarrow \deg _{s}\sum _{i=0}^{\infty }\alpha _{i}s^{i}=N . \end{aligned}$$

These distributions specify the marginal distributions of the dependent part (system II) of hierarchic first-order reaction networks. We will show in Sect. 3.3, that the $\text {DCP}^{N}$ and $\text {KTB}^{N}$ distributions are conditionally multimodal for $N>1$.

#### Proposition 3

(Marginal distributions given $\partial _{s_{X}}\lambda _{i}(s_{X})=0$) Let $\lambda _{i}(s_{X})$ be the *i*th eigenvalue of ${\mathbf {J}}(s_{X},t',t)$. Furthermore, let $\partial _{s_{X}}\lambda _{i}(s_{X})=0$, ${\mathbf {f}}={\mathbf {0}}$ and ${\mathbf {b}}={\mathbf {0}}$. Given Poissonian initial conditions, the marginal distribution of any species *X* from the dependent part of the network is $\text {DCP}^{N}$, where $N<\infty$. Given deterministic initial conditions, this distribution is $\text {KTB}^{N}$.

To prove this statement, we need an auxiliary result:

#### Lemma 2

Let ${\mathbf {H}}(x)$ be an $n\times n$ matrix, whose entries depend polynomially on $x\in {\mathbb {C}}$. Let $\lambda _{i}(x)$ be the *i*th eigenvalue of ${\mathbf {H}}(x)$. Then, $\partial _{x}\lambda _{i}(x)=0\Rightarrow \deg _{x}\big (e^{{\mathbf {H}}(x)}\big )_{ij}<\infty$.

#### Proof

By the Cayley–Hamilton theorem, ${\mathbf {H}}(x)$ fulfills its own characteristic polynomial $\varDelta (\lambda )$, i.e. $\varDelta ({\mathbf {H}}(x))=0$. Furthermore, any polynomial $p(\lambda )$ might be expressed as $p(\lambda )=q(\lambda )\varDelta (\lambda )+r(\lambda )$, where *q* is found by long division *p* / *q* with remainder *r* of degree $\le n-1$. Since $\varDelta ({\mathbf {H}}(x))={\mathbf {0}}$, we might express^9^ $e^{{\mathbf {H}}(x)}$ as

26 $$\begin{aligned} \underbrace{e^{{\mathbf {H}}(x)}}_{p({\mathbf {H}}(x))}=\underbrace{\sum _{k=0}^{n-1}\alpha _{k}(x){\mathbf {H}}^{k}(x)}_{r({\mathbf {H}}(x))} \end{aligned}$$

for some coefficients $\alpha _{k}(x)$.

Furthermore, the eigenvalues $\lambda _{i}(x)$ fulfill

27 $$\begin{aligned} e^{\lambda _{i}(x)}=\sum _{k=0}^{\infty }\frac{\lambda _{i}^{k}(x)}{k!}=\underbrace{\sum _{k=0}^{n-1}\alpha _{k}(x)\lambda _{i}^{k}(x)}_{:=\big ({\mathbf {L}}\alpha \big )_{i}}\qquad (i\in \{1,\ldots ,n\}). \end{aligned}$$

Here, the second relation follows again from long division by the characteristic polynomial of ${\mathbf {H}}(x)$. For $\partial _{x}\lambda _{i}(x)=0$, the coefficients of the characteristic polynomial do not depend on *x* and thus $\partial _{x}\alpha _{k}(x)=0$.

Therefore, the expression (26) introduces only a finite-order dependence on *x*. $\square$

Using this Lemma, we prove Proposition 3:

#### Proof

We obtain the marginal distribution of species *X* by setting all $s_{i}=1$ except $s_{X}$ in Eqs. (19) and (23) respectively. First of all, the integrals in Eq. (19) do not change the order of the polynomial in the exponent with respect to $s_{X}$, that is $\deg _{s_{X}}\big (e^{-{\mathbf {J}}(s_{X},t,t')}\big )_{ij}=\deg _{s_{X}}\big (e^{-\int _{0}^{t}{\mathbf {J}}(s_{X},t',t)dt'}\big )_{ij}$. Therefore, we have a $\text {DCP}^{N}$ distribution, where *N* is given by (24). We find^10^

28 $$\begin{aligned} N=\deg _{s_{X}}\left( {\mathbf {s}}_{\text {ind}}^{0}(s_{X})\cdot \big<{\mathbf {x}}_{{\mathbf {1}}}\big>_{0}\right)&=\deg _{s_{X}}\left( e^{-{\mathbf {J}}(s_{X})}{\mathbf {1}}\cdot \big <{\mathbf {x}}_{{\mathbf {1}}}\big >_{0}\right) =\deg _{s_{X}}\sum _{i,j}\left( e^{-{\mathbf {J}}(s_{X})}\right) _{ij} \end{aligned}$$

29 $$\begin{aligned}&\le \max _{i,j\in \{1,\ldots ,n_{\text {ind}}\}}\deg _{s_{X}}\left( e^{-{\mathbf {J}}(s_{X})}\right) _{ij}. \end{aligned}$$

Because ${\mathbf {J}}(s_{X})$ depends polynomially on $s_{X}$, we apply Lemma 2 to obtain

$$\begin{aligned} N\le \deg _{s_{X}}\left( e^{-{\mathbf {J}}(s_{X})}\right) _{ij}<\infty . \end{aligned}$$

In case $[{\mathbf {J}}(s_{X},t_{1},t),{\mathbf {J}}(s_{X},t_{2},t)]\not ={\mathbf {0}}$, the same argument can be made for the Magnus series. By replacing $\big <{\mathbf {x_{1}}}\big >_{0}$ in Eq. (28) with $\varepsilon _{i}^{(1)}:=\text {col}_{i}{\mathbf {I}}_{n_{\text {ind}}\times n_{\text {ind}}}$ for deterministic initial conditions, we have the result $\text {KTB}^{N}$, where $N<\infty$. $\square$

For simple reaction networks such as those from the next proposition, we show the order of the marginal distributions to be larger than one. We first investigate networks whose independent part is mass-conservative, i.e. $\sum _{l=1}^{n_{\text {ind}}}R_{li}\not =0\Rightarrow {\mathbf {f}}={\mathbf {0}}$ and $\sum _{l=1}^{n_{\text {ind}}}Q{}_{li}\not ={0}\Rightarrow {\mathbf {b}}={\mathbf {0}}$.

#### Proposition 4

Let $\partial _{s_{X}}\lambda _{i}(s_{X})=0$, ${\mathbf {f}}={\mathbf {0}}$, ${\mathbf {b}}={\mathbf {0}}$ and $[{\mathbf {J}}(s_{X},t_{1},t),{\mathbf {J}}(s_{X},t_{2},t)]={\mathbf {0}}$. The marginal distribution of any dependent species *X* is $\text {DCP}^{N}$ ($\text {KTB}^{N}$ for deterministic initial conditions), with $N>1$ for the following minimal reaction networks:

1. $$\begin{aligned} S_{1}^{\text {ind}}\rightarrow S_{2}^{\text {ind}}+RX,\qquad (\text {Type I}) \end{aligned}$$
  where $$R>1$$ and $$n_{\text {ind}}=2$$.
2. $$\begin{aligned} \begin{array}{cc} S_{1}^{\text {ind}}\rightarrow &{} S_{2}^{\text {ind}}+R_{1}X\\ S_{2}^{\text {ind}}\rightarrow &{} S_{3}^{\text {ind}}+R_{2}X, \end{array}\qquad (\text {Type II}) \end{aligned}$$
  where $$R_{1}\ge 1$$, $$R_{2}\ge 1$$ and $$n_{\text {ind}}=3$$.

#### Proof

We expand the matrix powers in

30 $$\begin{aligned} \deg _{s_{X}}\sum _{p,q}(e^{-{\mathbf {J}}(s_{X})})_{pq}{\mathop {=}\limits ^{(26)}}\deg _{s_{X}}\sum _{p,q}\Bigg (\sum _{k=0}^{n_{\text {ind}}-1} (-\mathbf {J}(s_X))^k \alpha _k \Bigg )_{pq}, \end{aligned}$$

starting with $${\mathbf {J}}^{1}$$:

$$\begin{aligned} J_{pq}(s_{X}){\mathop {=}\limits ^{(20)}}\sum \limits _{i=1}^{m}k_{i}Q_{pi}(1-c_{i}(s_{X})R_{qi}),\quad p,q\in \{1,\ldots ,n_{\text {ind}}\} \end{aligned}$$

For reaction Type I, it suffices to consider the first order ${\mathbf {J}}^{1}$ of (30). Since there are no reactions within the dependent part ${\mathbf {A}}={\mathbf {0}}$, we then have

31 $$\begin{aligned} c_{i}(s_{X},t',t){\mathop {=}\limits ^{(22)}}&\Big (1\cdot (s_{X}-1)+1\Big )^{R_{i}}=s_{X}^{R_{i}} \end{aligned}$$

In consequence of (31), we get $\deg _{s_{X}}\sum _{p,q}(e^{{\mathbf {J}}(s_{X})})_{pq}>1$ whenever $R_{i}>1$. This translates to a splitting reaction of Type I. To see $\partial _{s_{X}}\lambda _{i}(s_{X})=0$, consider

$$\begin{aligned} \frac{ds_{1}}{dt}&=-\underbrace{k_{S_{1}\rightarrow S_{2}+X}}_{:=1}(s_{X}^{R}s_{2}-s_{1})\\ \frac{ds_{2}}{dt}&=0 \end{aligned}$$

and

$$\begin{aligned} \det ({\mathbf {J}}-\lambda {\mathbf {1}})=\det ((\partial _{s_{j}}\frac{ds_{i}}{dt})_{ij}-\lambda {\mathbf {1}})=\det \left( \begin{array}{cc} 1-\lambda &{} -s_{X}^{R}\\ 0 &{} -\lambda \end{array}\right) =\lambda ^{2}-\lambda . \end{aligned}$$

For reaction Type II, $n_{\text {ind}}=3$ and we need to calculate the second order ${\mathbf {J}}^{2}$ in (30). We have^11^

$$\begin{aligned} J_{pq}^{2}(s_{X})&\propto \sum _{r=1}^{n_{\text {ind}}}\left( \sum \limits _{i=1}^{m}k_{i}Q_{pi}(1-s_{X}^{R_{i}}R_{ri})\right) \left( \sum \limits _{j=1}^{m}k_{j}Q_{rj}(1-s_{X}^{R_{j}}R_{qj})\right) \\&\propto \sum _{r=1}^{n_{\text {ind}}}\sum \limits _{i,j=1}^{m}k_{i}k_{j}Q_{pi}R_{ri}Q_{rj}R_{qj}s_{X}^{R_{i}+R_{j}}. \end{aligned}$$

Note that only $i\not =j$ terms are non-zero due to $\sum _{l=1}^{n_{\text {ind}}}R_{li}\le 1$. This implies that the products from the independent part must appear in different reactions. In terms of chemical reaction networks, we have

$$\begin{aligned} Q_{p1}S_{p}^{\text {ind}}&\rightarrow R_{r1}S_{r}^{\text {ind}}+R_{\xi 1}X\\ Q_{r2}S_{r}^{\text {ind}}&\rightarrow R_{q2}S_{q}^{\text {ind}}+R_{\xi 2}X. \end{aligned}$$

We arbitrarily chose $p=1$, $r=2$, $q=3$ to obtain the result. To see $\partial _{s_{X}}\lambda _{i}(s_{X})=0$, consider

$$\begin{aligned} \frac{ds_{1}}{dt}&=-\underbrace{k_{S_{1}\rightarrow S_{2}+R_{1}X}}_{:=1}(s_{X}^{R_{1}}s_{2}-s_{1})\\ \frac{ds_{2}}{dt}&=-\underbrace{k_{S_{2}\rightarrow S_{3}+R_{2}X}}_{:=1}(s_{X}^{R_{2}}s_{3}-s_{2})\\ \frac{ds_{3}}{dt}&=0 \end{aligned}$$

and

$$\begin{aligned} \det {\mathbf {J}}=\det (\partial _{s_{j}}\frac{ds_{i}}{dt})_{ij}=\det \left( \begin{array}{ccc} 1-\lambda &{} -s_{X}^{R_{1}} &{} 0\\ 0 &{} 1-\lambda &{} -s_{X}^{R_{2}}\\ 0 &{} 0 &{} -\lambda \end{array}\right) =-\lambda \left( \lambda ^{2}-2\lambda +1\right) . \end{aligned}$$

$$\square $$

#### Proposition 5

Let ${\mathbf {J}}(t',t)$ be independent of $s_{X}$, ${\mathbf {b}}={\mathbf {0}}$ and $\partial _{s_{X}}{\mathbf {f}}(s_{X},t',t)\not ={\mathbf {0}}$, then the marginal distribution of any species *X* from the dependent part is $\text {DCP}^{N}$ ($\text {KTB}^{N}$ for deterministic initial conditions), with $N>1$ for the following minimal reaction network:

$$\begin{aligned} S_{1}^{\text {ind}}\rightarrow RX, \end{aligned}$$

where $$R>1$$ and $$n_{\text {ind}}=1$$.

#### Proof

We express the reaction $S_{1}^{\text {ind}}\rightarrow RX$ by

$$\begin{aligned} f_{1}(s_{X}){\mathop {=}\limits ^{(21)}}-k_{1}\Big (1\cdot (s_{X}-1)+1\Big )^{R}=-k_{1}s_{X}^{R} \end{aligned}$$

and plug the same expression into (19) to obtain $\log g(s_{X},t)\propto s_{X}^{R}$. In consequence, we get a $\text {DCP}^{N}$ distribution with $N>1$ whenever $R>1$. The analogous statement for deterministic initial conditions follows by plugging $f_{1}(s_{X})$ into (23). $\square$

The next proposition holds for the transcription–translation model, as examined in detail in Example 5.

#### Proposition 6

(Marginal distributions given $\partial _{s_{X}}\lambda _{i}(s_{X})\not =0$) Let $\lambda _{i}(s_{X})$ be the *i*th eigenvalue of ${\mathbf {J}}(s_{X},t',t)$ and let $\partial _{s_{X}}\lambda (s_{X})\not =0$. Given Poissonian initial conditions, the marginal distribution of any species *X* from the dependent part of the network is $\text {DCP}^{\infty }$ ($\text {KTB}{}^{\infty }$ for deterministic initial conditions).

#### Proof

In case all eigenvalues are distinct, we solve the system (27) by inverting the matrix^12^ ${\mathbf {L}}(s_{X})$, so

32 $$\begin{aligned} e^{{\mathbf {J}}(s_{X})}=\sum _{k=0}^{n_{\text {ind}}-1}\sum _{i=0}^{n_{\text {ind}}-1}({\mathbf {L}}^{-1}(s_{X}))_{ki}e^{\lambda _{i}(s_{X})}{\mathbf {J}}^{k}(s_{X}). \end{aligned}$$

Since $({\mathbf {L}}^{-1})_{ki}$ is a rational function of the eigenvalues, we have

33 $$\begin{aligned} \max _{i,j\in \{1,\ldots ,n_{\text {ind}}\}}\deg _{s_{X}}\big (e^{{\mathbf {J}}(s_{X})}\big )_{ij}=\infty . \end{aligned}$$

In case there is an eigenvalue $\lambda _{j}$ of multiplicity $\mu$, the matrix ${\mathbf {L}}$ is not invertible. Since

$$\begin{aligned} \frac{d^{\mu -1}\varDelta (\lambda )}{d\lambda ^{\mu -1}}\Bigg |_{\lambda _{j}}=\frac{d^{\mu -1}}{d\lambda ^{\mu -1}}(\lambda -\lambda _{j})^{\mu }\prod _{i=1}^{n_{\text {ind}}-\mu }(\lambda -\lambda _{i})\Bigg |_{\lambda _{j}}=0\ , \end{aligned}$$

we derive (27) for $\lambda$ and obtain $\mu -1$ additional equations:

$$\begin{aligned} \frac{d^{i}e^{\lambda _{j}(s_{X})}}{d\lambda ^{i}}=\frac{d^{i}}{d\lambda ^{i}}\sum _{k=0}^{n_{\text {ind}}-1}\alpha _{k}(s_{X})\lambda ^{k}\qquad (i\in \{1,\ldots ,\mu -1\}). \end{aligned}$$

We solve this system together with (27) for $\alpha _{k}(s_{X})$ by inverting a matrix. The entries of this matrix are rational functions of $\lambda$, so the terms $e^{\lambda _{i}(s_{X})}$ in (32) do not cancel, i.e. Equation (33) holds. In the last step, we plug Eq. (32) into (19) for Poissonian and into (23) for deterministic initial conditions to obtain the generating function for the marginal distribution of *X*. Since the exponent of (19) is of infinite degree, we obtain a $\text {DCP}^{\infty }$ class distribution. For deterministic initial conditions, we obtain a $\text {KTB}^{\infty }$ class distribution. $\square$

### Modality of marginal distributions

In this section, we investigate under which conditions the generalized distributions from Proposition 2 have infinitely many modes. We need several definitions for this end:

#### Definition 7

(*Unimodality*) A distribution $p_{n}$ is said to be *unimodal* with mode $a\in {\mathbb {N}}_{\text {0}}$ if

$$\begin{aligned} p_{n}{\ge }p_{n-1},\ \forall \ n\le a\quad \text {and}\quad p_{n+1}\le p_{n},\ \forall \ n\ge a. \end{aligned}$$

If this property does not depend on the parameters of the distribution, the latter is said to be *unconditionally unimodal*. A unimodal distribution that results in a unimodal distribution upon convolution with another unimodal distribution is called *strongly unimodal*.^13^

The classes $\text {DCP}^{1}$ and $\text {KTB}^{1}$ are unconditionally unimodal, since the only members of these sets are the Poissonian and Binomial distribution respectively. For the independent species (system I) of a hierarchic first-order reaction network, we obtain unimodal marginal distributions:

#### Theorem 2

(Unconditional unimodality of marginal distributions of independent species (system I)) Given deterministic or Poissonian initial conditions, the species from the independent part of the hierarchic network exhibit unconditionally unimodal marginal distributions at all times $t>0$.

#### Proof

Upon setting all $s_{i}=1$, except one $s_{X}$ from the independent part, we obtain a Poissonian distribution for Eq. (19). For deterministic initial conditions, Eq. (23) yields a convolution of binomial and Poisson distributions. Since the binomial and Poisson distribution are strongly unimodal, the resulting distribution is unimodal as well, as shown by Keilson and Gerber (1971). $\square$

For the second part of a hierarchic first-order network, we need the following definition:

#### Definition 8

(*Conditional multimodality*) A distribution that is not unconditionally unimodal is called *conditionally multimodal*.

As an example for the preceding definition, we have Neyman’s Type A distribution (Neyman 1939), defined by the generating function $\exp (\lambda (e^{\phi (s-1)}-1))$, where $\lambda >0$ and $\phi >0$. Because of the double exponential generation function, it belongs to the class of $\text {DCP}^{\infty }$ distributions that we prove to be conditionally multimodal in the next proposition.

#### Proposition 7

(Three classes of conditionally multimodal distributions) The classes of distributions $\text {DCP}^{\infty }$, $\text {KTB}^{\infty }$, $\text {DCP}^{2}$, $\text {KTB}^{2}$ and $\text {DCP}^{3}$, $\text {KTB}^{3}$ are conditionally multimodal.

#### Proof

**Fig. 2:**
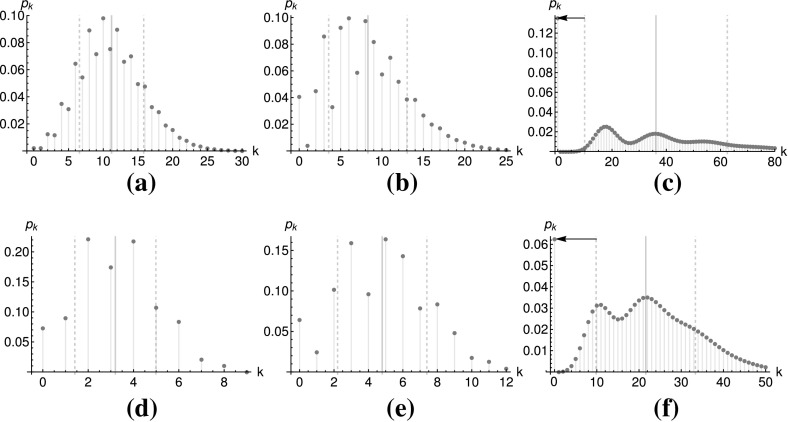
Some conditionally multimodal distributions. The thick gray lines depict the expectation value, while the dashed lines stand for the standard deviation. The arrow marks the probability for a zero outcome. **a** Hermite ($\text {DCP}^{2}$) distribution, $a_{1}=1$, $a_{2}=5.1$. **b** Triple stuttering-Poisson ($\text {DCP}^{3}$) distribution, $a_{1}=0.1$, $a_{2}=1.1$, $a_{3}=2$. **c** Neyman’s type A ($\text {DCP}^{\infty }$) distribution, $\lambda =2$, $\phi =18.1$. **d** Binomial–Binomial ($\text {KTB}^{2}$) distribution, $p_{1}=0.5$, $p_{2}=0.8$. **e** Binomial–Binomial ($\text {KTB}^{3}$) distribution, $p_{1}=0.5$, $p_{2}=0.8$. **f** Binomial–Binomial ($\text {KTB}^{infinity}$) distribution, $p_{1}=0.5$, $p_{2}=0.8$

Since conditional multimodality is defined to be the contrary of unconditional unimodality, a counterexample is enough to prove conditional multimodality. Respective counterexamples are shown in Fig. 2. Figure 2a shows Neyman’s Type A distribution, defined by the $\text {DCP}^{\infty }$ generating function $g(s)=\exp (\lambda (e^{\phi (s-1)}-1))$, can be multimodal.^14^ The same is done for the Hermite distribution by the generating function $g(s)=e^{a_{1}(s-1)+a_{2}(s^{2}-1)}$. As a $\text {DCP}^{2}$ distribution, it is conditionally multimodal, as demonstrated in Fig. 2a. A counterexample for $\text {DCP}^{3}$ is given by $g(s)=e^{a_{1}(s-1)+a_{2}(s^{2}-1)+a_{3}(s^{3}-1)}$ from Fig. 2b. Figure 2d–f shows counterexamples for the generalized binomial distributions $\text {KTB}^{2}$, $\text {KTB}^{3}$ and $\text {KTB}^{\infty }$:

$$\begin{aligned} g_{\text {KTB}^{2}}(s)&=\big (1-p_{1}+p_{1}(1-p_{2}+p_{2}s)^{2}\big )^{4}&\big (\text {Binomial}(4,p_{1})\bigvee \text {Binomial}(2,p_{2})\big )\\ g_{\text {KTB}^{3}}(s)&=\big (1-p_{1}+p_{1}(1-p_{2}+p_{2}s)^{3}\big )^{4}&\big (\text {Binomial}(4,p_{1})\bigvee \text {Binomial}(3,p_{2})\big )\\ g_{\text {KTB}^{\infty }}(s)&=\big (1-p_{1}+p_{1}e^{\lambda (s-1)}\big )^{4}&\big (\text {Binomial}(4,p_{1})\bigvee \text {Poisson}(\lambda )\big ). \end{aligned}$$

$$\square $$

Since we cannot provide an infinite number of counterexamples, one for each $\text {DCP}^{N}$ or $\text {KTB}^{N}$ class, where $N>1$, we extend the result of the last proposition as a conjecture.

#### Conjecture 1

The classes $\text {DCP}^{N}$ and $\text {KTB}^{N}$ are conditionally multimodal for $N>3$.

The intuition behind this conjecture is that the $\text {DCP}{}^{N}$ and $\text {KTB}^{N}$ classes are modeling events that can occur in bursts. In terms of Definition 4, the generalizing distribution ${\mathscr {P}}_{2}$ is modeling the (stochastic) number of events per burst or burst size, whereas the generalized distribution ${\mathscr {P}}_{1}$ models the (stochastic) number of bursts, yielding the total number of events as ${{\mathscr {P}}\sim {\mathscr {P}}_{1}\bigvee {\mathscr {P}}_{2}}$. In consequence, the distances between the modes seen in Fig. 2 approximately correspond to multiples of the burst sizes. For the Binomial–Binomial distribution from Fig. 2d the burst size is 2, for Fig. 2e it is 3, and for the Binomial–Poisson distribution from Fig. 2f it is $\lambda =10.8$. The same applies for the generalized Poisson distributions. For Neyman’s Type A (Poisson–Poisson) distribution shown in Fig. 2c, the modes are clearly visible at multiples of 18.1. Thus, the bursting behavior is linked to conditional multimodality. In consequence, what we are conjecturing is that this link stays the same as we increase the number of possible bursts beyond 3.

In the next Theorems, we summarize the cases in which conditional multimodality appears for the species of the dependent system II.

#### Theorem 3

(Sufficient criterion for conditional multimodality, given deterministic or Poissonian initial conditions and $\partial _{s_{X}}\lambda (s_{X})\not =0$) Let $\partial _{s_{X}}\lambda (s_{X})\not =0$. Then the dependent species *X* in a hierarchic first-order network obeys a conditionally multimodal marginal distribution at all times $t>0$.

#### Proof

$\partial _{s_{X}}\lambda (s_{X})\not =0$ implies $\text {DCP}^{\infty }$ and $\text {KTB}^{\infty }$ class distributions (Proposition 6) that are conditionally multimodal by Proposition 7. $\square$

#### Theorem 4

(Minimal reaction networks exposing conditional multimodality, given deterministic or Poissonian initial conditions and $\partial _{s_{X}}\lambda (s_{X})=0$) Let $\partial _{s_{X}}\lambda (s_{X})=0$, ${\mathbf {b}}={\mathbf {0}}$ and assume Conjecture 1 is true. Then, given Poissonian or deterministic initial conditions, the following minimal hierarchic first-order networks exhibit a conditionally multimodal marginal distribution at all times $t>0$ for the dependent species X:

1. For mass-conservative independent networks, i.e. ${\mathbf {f}}={\mathbf {0}}$:
  - $$\begin{aligned} S_{1}^{\text {ind}}\rightarrow S_{2}^{\text {ind}}+RX,\qquad (\text {Type I}) \end{aligned}$$
    where $$R>1$$ and $$n_{\text {ind}}=2$$.
  - $$\begin{aligned} \begin{array}{cc} S_{1}^{\text {ind}}\rightarrow &{} S_{2}^{\text {ind}}+R_{1}X\\ S_{2}^{\text {ind}}\rightarrow &{} S_{3}^{\text {ind}}+R_{2}X, \end{array}\qquad (\text {Type II}) \end{aligned}$$
    where $$R_{1}\ge 1$$, $$R_{2}\ge 1$$ and $$n_{\text {ind}}=3$$.
2. For open independent networks and $\sum _{l=1}^{n_{\text {ind}}}R_{li}=0$ for all reactions, i.e. $\partial _{s_{X}}{\mathbf {J}}={\mathbf {0}}$ and $\partial _{s_{X}}{\mathbf {f}}\not ={\mathbf {0}}$, the minimal network is:
  $$\begin{aligned} S_{1}^{\text {ind}}\rightarrow RX \end{aligned}$$
  where $$R>1$$ and $$n_{\text {ind}}=1$$.

#### Proof

By Proposition 4 we know that the distribution class is $\text {DCP}^{N_{\mathrm{Prop4}}}$ whenever ${\mathbf {f}}={\mathbf {0}}$, where the degree $N_{\mathrm{Prop4}}>1$ for both types of minimal reaction networks. Proposition 5 applies if $\partial _{s_{X}}{\mathbf {f}}\not ={\mathbf {0}}$, and yields the minimal reaction network $S_{1}^{\text {ind}}\rightarrow RX$ for which $N_{\mathrm{Prop5}}>1$.

These distribution classes are conditionally multimodal if $N_{\mathrm{Prop4}}$ and $N_{\mathrm{Prop5}}$ are larger than one according to Proposition 7 and Conjecture 1. $\square$

In general conditional multimodality arises, if the degree of the exponent of (19) is larger than one upon setting all $s_{i}=1$ except $s_{X}$.

## Examples

In the following two sections, we illustrate how our results can be used to predict conditional multimodality by several examples. The calculations can be verified using the supplemented Wolfram Mathematica files.

### Basic models

#### Example 1

(Catalysis) The model of the simple catalytic reaction $X{\mathop {\rightarrow }\limits ^{k_{\text {cat}}}}X+Y$ is a trivial example of a hierarchic system, since system II is governed by a time-independent characteristic ODE. By Eqs. (6–7) we have

34 $$\begin{aligned} \frac{ds_{X}}{dt}&=-k_{\text {cat}}(s_{X}s_{Y}-s_{X}) \end{aligned}$$

35 $$\begin{aligned} \frac{ds_{Y}}{dt}&=0 \end{aligned}$$

36 $$\begin{aligned} \frac{dg}{dt}&=0\Leftrightarrow g=g(s_{X}^{0},s_{Y}^{0},0). \end{aligned}$$

The solution of (35) simply reads $s_{Y}(t)=s_{Y}^{0}$. We reorder the terms in (34)

$$\begin{aligned} \frac{ds_{X}}{dt}= & {} s_{X}(-k_{\text {cat}}(s_{Y}^{0}-1)), \end{aligned}$$

and solve the same equation as

$$\begin{aligned} s_{X}(t)= & {} e^{-k_{\text {cat}}t(s_{Y}^{0}-1)}s_{X}^{0}. \end{aligned}$$

The generating function, given by (8) for Poissonian initial conditions, reads

$$\begin{aligned} g_{\text {Poiss}}(s_{X}^{0},s_{Y}^{0},0)= & {} \exp \Big (\big<x\big>_{0}(s_{X}^{0}-1)+\big <y\big >_{0}(s_{Y}^{0}-1)\Big )\ . \end{aligned}$$

By replacing

$$\begin{aligned} s_{X}^{0}=e^{k_{\text {cat}}t(s_{Y}^{0}-1)}s_{X}, \end{aligned}$$

and $s_{Y}=s_{Y}^{0}$ we obtain the generating function

37 $$\begin{aligned} g_{\text {GenPoiss}}(s_{X},s_{Y},t)= & {} \exp \Big (\big<x\big>_{0}(e^{k_{\text {cat}}t(s_{Y}-1)}s_{X}-1)+\big <y\big >_{0}(s_{Y}-1)\Big )\ . \end{aligned}$$

The marginal distribution of *Y* can be represented as a convolution of Neyman’s type A distribution and a Poisson distribution:

38 $$\begin{aligned} {\mathscr {P}}\sim \big [\underbrace{\text {Poisson}(\big<x\big>_{0})\bigvee \text {Poisson}(k_{\text {cat}}t)}_{\text {Neyman Type A}}\big ]*\text {Poisson}(\big <y\big >_{0}). \end{aligned}$$

**Fig. 3:**
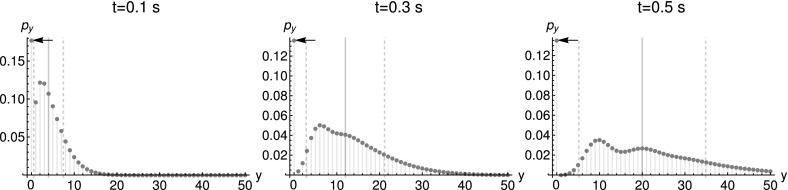
Marginal distributions for catalysis product $p_{y}:=P(y,t|y_{0}=0,0)$, given the parameters $k_{\text {cat}}=20~\text {s}^{-1}$, deterministic ($y_{0}=0$) and Poissonian initial conditions for $\big <x\big >_{0}=2$. The thick gray lines mark the expectation value, and the dashed lines the range of one standard deviation from the mean. The arrow marks the probability for a zero outcome

In case we set $y_{0}=0$ as a deterministic initial condition, the distribution is identical to Neyman’s Type A distribution. In Fig. 3, we depict a numerical evaluation of this multimodal distribution. Conditional multimodality is expected by Theorem 3, since the Jacobian exhibits eigenvalues dependent on $s_{Y}$. Note that the most likely outcome is zero, is far from the mean (solid gray line in Fig. 3), even outside the standard deviation (dashed gray line). This clearly demonstrates the misleading character of mean and standard deviation for multimodal distributions. The high probability for a zero outcome, depicted by the black arrow in Fig. 3, can be explained by the high likelihood of an “extinction scenario”: If there are initially no molecules of species *X*, the number of molecules of type *Y* must always be zero. This phenomenon can be found in many hierarchic networks, as we show in the following figures.

Using deterministic initial conditions, we obtain a contrasting picture, since the resulting marginal distributions are Poissonian:

39 $$\begin{aligned} g_{\text {det}}(s_{X},s_{Y},t)&=(s_{X})^{x_{0}}(s_{Y})^{y_{0}}=(e^{k_{\text {cat}}t(s_{Y}-1)}s_{X})^{x_{0}}(s_{Y})^{y_{0}}\nonumber \\ \Rightarrow g_{\text {det}}(1,s_{Y},t)&=\underbrace{(e^{k_{\text {cat}}t(s_{Y}-1)})^{x_{0}}}_{:=g_{\text {cat}}(s_{Y})}. \end{aligned}$$

**Fig. 4:**
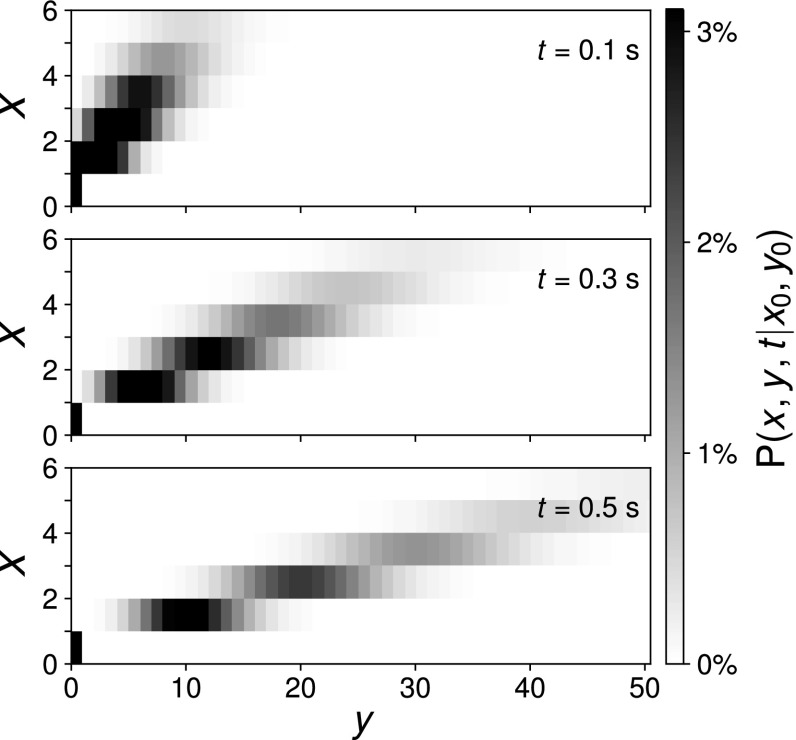
Plot of the joint distribution for the educt *X* and product *Y* for the same parameters and time points as Fig. 3

In consequence, no multimodality is observed. However, since ${\mathscr {P}}_{\text {cat}}\in \text {KTB}^{\infty }$, Proposition 7 predicts conditionally multimodality. This is not a contradiction because conditional multimodality is a notion defined by the order of the generalizing distribution $g_{2}(s)$ (see Def. 6), not by the model parameters (here $k_{\text {cat}}$). For a concrete model, only subsets of the parameter space, defined by the set of coefficients of $g_{2}(s)$, are reachable by appropriate choices of the model parameters (here $k_{\text {cat}}$). In our model this subset does not contain to multimodal distributions. The joint distribution is plotted in Fig. 4.

To obtain a multimodal distribution for deterministic initial conditions, we add a degradation reaction, as shown in the next example.

#### Example 2

(Catalysis with degradation) We see a different picture for deterministic initial conditions once we add a degradation mechanism:

$$\begin{aligned} X{\mathop {\rightarrow }\limits ^{k_{\text {cat}}}}&X+Y\\ X{\mathop {\rightarrow }\limits ^{k_{\text {deg}}}}&\emptyset . \end{aligned}$$

These reactions result in the characteristic system

$$\begin{aligned} \frac{ds_{X}}{dt}&=-\,k_{\text {cat}}(s_{X}s_{Y}-s_{X})-k_{\text {deg}}(1-s_{X})\\ \frac{ds_{Y}}{dt}&=0, \end{aligned}$$

solved by

$$\begin{aligned} s_{X}(t)=\, \frac{\big (s_{X}(k_{\text {cat}}(1-s_{Y})+k_{\text {deg}})-k_{\text {deg}}\big )e^{t(k_{\text {cat}}(1-s_{Y})+k_{\text {deg}})}+k_{\text {deg}}}{k_{\text {cat}}(1-s_{Y})-k_{\text {deg}}}\ . \end{aligned}$$

**Fig. 5:**
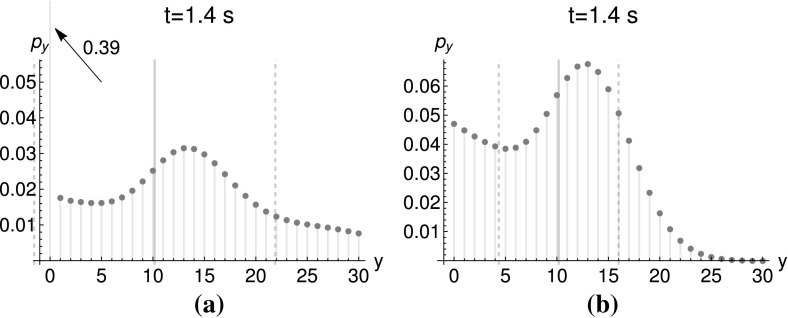
Marginal distributions $p_{y}:=P(y,t|y_{0}=0,0)$ at $t=1.4~\hbox {s}$. Parameters: $k_{\text {cat}}=10.1~\hbox {s}^{-1}$, $k_{\text {deg}}=0.5~\hbox {s}^{-1}$. The thick gray lines depict the expectation value, while the dashed lines stand for the standard deviation. The arrow marks the probability for a zero outcome. **a** Poissonian initial conditions: $\big <x\big >_{0}=1$, ${\big <y\big >_{0}=0.001}$. **b** Deterministic initial conditions: $x_{0}=1$, $y_{0}=0$

Deterministic initial conditions result in the following generating function:

$$\begin{aligned}&g(s_{X},s_{Y},t)\\&\quad = \Bigg (\frac{e^{-t(k_{\text {cat}}(1-s_{Y})+k_{\text {deg}})}\left( -s_{X}(k_{\text {cat}}(1-s_{Y})+k_{\text {deg}})+k_{\text {deg}}\left( -e^{t(k_{\text {cat}}(1-s_{Y})+k_{\text {deg}})}\right) +k_{\text {deg}}\right) }{k_{\text {cat}}(s_{Y}-1)-k_{\text {deg}}}\Bigg )^{x_{0}}(s_{Y})^{y_{0}}. \end{aligned}$$

Figure 5 shows the comparison between Poissonian and deterministic initial conditions. For Poissonian initial conditions, the scenario of starting without any molecules of species *X* is possible. This “extinction scenario” does not lead to the production of any *Y* molecules at any time and is seen by the large peak at $y=0$ in Fig. 5a. For deterministic initial conditions, we set $x_{0}=1$ and Fig. 5b therefore does not exhibit a peak of the same size at $y=0$. However, after some time, the species *X* may well become extinct and therefore the mode at $y=0$ still appears, albeit with a lower size compared to Fig. 5a.

#### Example 3

(Simple splitting) The simple splitting reaction $X{\mathop {\rightarrow }\limits ^{k_{1}}}Y+Z$, obeys the characteristic system

$$\begin{aligned} \frac{ds_{X}}{dt}&=-\,k_{1}(s_{Y}s_{Z}-s_{X})\\ \frac{ds_{Y}}{dt}&=0\\ \frac{ds_{Z}}{dt}&=0. \end{aligned}$$

It is solved by

$$\begin{aligned} s_{X}(t)&=s_{X}^{0}e^{k_{1}t}+s_{Y}s_{Z}(1-e^{k_{1}t})\\ \Leftrightarrow s_{X}^{0}&=e^{-k_{1}t}s_{X}(t)-s_{Y}s_{Z}(e^{-k_{1}t}-1). \end{aligned}$$

Poissonian initial conditions yield the generating function

40 $$\begin{aligned}&g(s_{X},s_{Y},s_{Z},t)\nonumber \\&\quad = \exp \Big \{\big<x_{1}\big>_{0}(e^{-k_{1}t}s_{X}(t)-s_{Y}s_{Z}(e^{-k_{1}t}-1)-1)+\big<x_{2}\big>_{0}(s_{Y}-1)+\big <x_{3}\big >_{0}(s_{Z}-1)\Big \}. \end{aligned}$$

Therefore, the marginal distributions are Poissonian and the joint distribution is a multivariate Poisson distribution, studied in detail by Kawamura (1979). The product Poisson distribution differs from the multivariate Poisson distribution by the fact that the variables *Y* and *Z* correlate, yet the marginal distributions stay unimodal for both.

Without the explicit solution (40), we prove unconditional unimodality by noting that either *Y* or *Z* may be assigned to the independent part of the network. In consequence, Theorem 2 can be applied to both *Y* and *Z*.

#### Example 4

(Additional conversion) Things get more interesting as we add a conversion reaction to the same network:

$$\begin{aligned} X&{\mathop {\rightarrow }\limits ^{k_{1}}}Y+Z\\ Y&{\mathop {\rightarrow }\limits ^{k_{2}}}X. \end{aligned}$$

The characteristic system is given by

$$\begin{aligned} \frac{ds_{X}}{dt}&=-\,k_{1}(s_{Y}s_{Z}-s_{X})\\ \frac{ds_{Y}}{dt}&=-\,k_{2}(s_{X}-s_{Y})\\ \frac{ds_{Z}}{dt}&=0, \end{aligned}$$

and the Jacobian by

$$\begin{aligned} {\mathbf {J}}(s_{Z})=\left( \begin{array}{cc} k_{1} &{} -k_{1}s_{Z}\\ -k_{2} &{} k_{2} \end{array}\right) . \end{aligned}$$

**Fig. 6:**
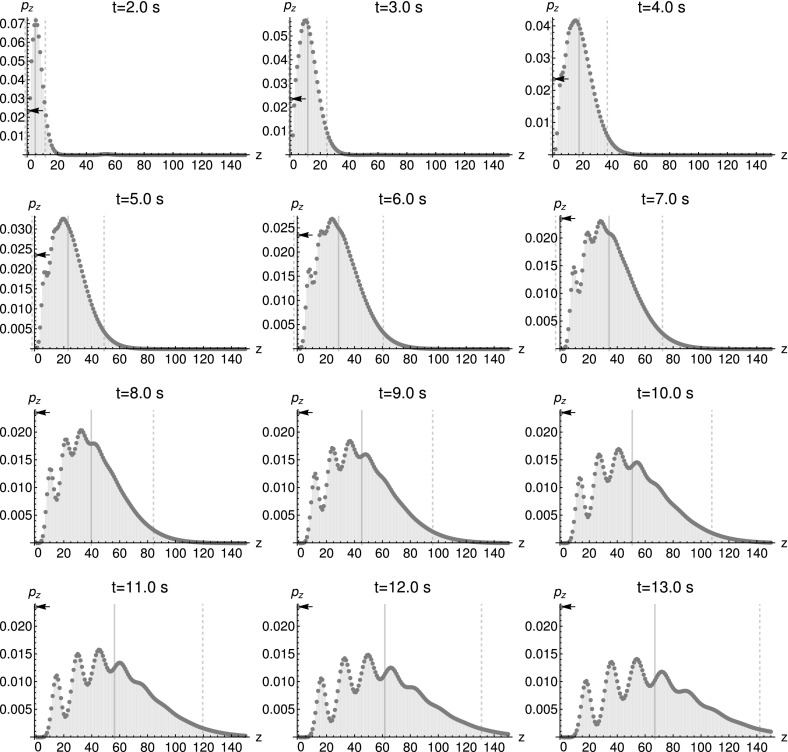
Marginal distribution $p_{z}:=P(z,t|z_{0}=0,0)$, given Poissonian initial conditions $\big <x\big >_{0}=1.875$, $\big <y\big >_{0}=1.875$, as well as the deterministic initial condition $\xi _{Z}=0$. Parameters are $k_{1}=7.0~\text {s}^{-1}$, $k_{2}=1.875~\text {s}^{-1}$. The thick black line represents the mean while the dashed lines depict the standard deviation. The arrow marks the probability for a zero outcome

**Fig. 7:**
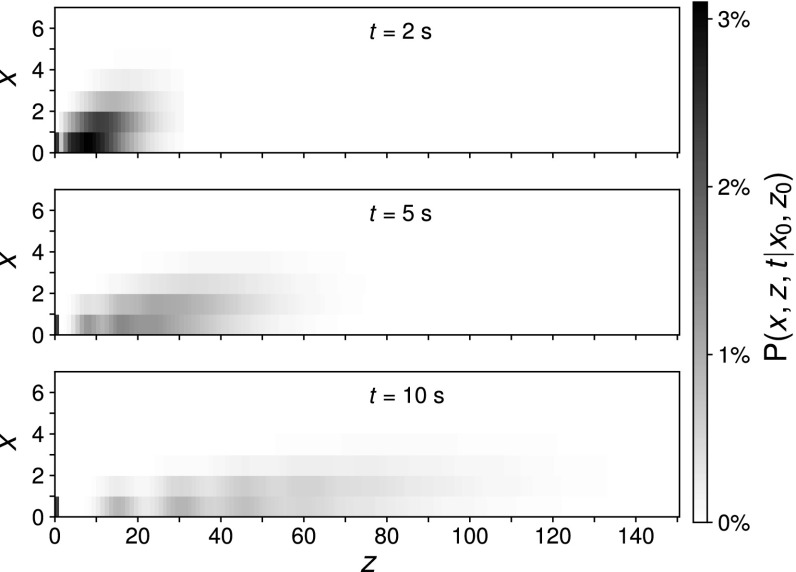
Plot of the joint distribution for the educt *X* and product *Z* for the same parameters as Fig. 6

The characteristic polynomial

$$\begin{aligned} \det (\lambda {\mathbf {I}}-\left( \begin{array}{cc} k_{1} &{} -k_{1}s_{Z}\\ -k_{2} &{} k_{2} \end{array}\right) )=-\,k_{1}\lambda -k_{2}\lambda -k_{1}k_{2}s_{Z}+k_{1}k_{2}+\lambda ^{2} \end{aligned}$$

depends on $s_{Z}$, which implies $\partial _{s_{Z}}\lambda (s_{Z})\not =0$. Proposition 6 therefore applies and the resulting distributions are conditionally multimodal. Multiple modes, obtained by assuming Poisson distributed initial numbers, are visible in the marginal distribution Fig. 6 as well as the joint distribution Fig. 7.

By adding a degradation reaction $X{\mathop {\rightarrow }\limits ^{k_{3}}}\emptyset$ to this model and setting $k_{2}\gg k_{1}$, we obtain a model that behaves like Example 2. In consequence, multimodal distributions appear for deterministic and Poissonian initial conditions for $k_{1}=k_{\text {cat}}$ and $k_{3}=k_{\text {deg}}$ (data not shown).

### Real world models

#### Example 5

(Transcription–translation model) The two-stage model, which excludes any effects of promoter activity, consists of the reactions

$$\begin{aligned} \emptyset&{\mathop {\rightarrow }\limits ^{k_{\text {mRNA}}}}X\\ X&{\mathop {\rightarrow }\limits ^{d_{\text {mRNA}}}}\emptyset \\ X&{\mathop {\rightarrow }\limits ^{k_{\text {tl}}}}X+Y\\ Y&{\mathop {\rightarrow }\limits ^{d_{\text {prot}}}}\emptyset , \end{aligned}$$

where *X* are mRNA molecules and *Y* proteins. We use the implementation of Gillespie’s algorithm by Maarleveld et al. (2013) to simulate this model.

**Fig. 8:**
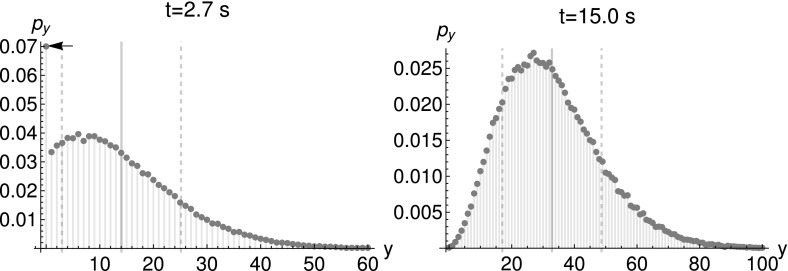
Marginal distribution of protein numbers $p_{y}:=P(y,t|y_{0}=0,0)$ created from $10^{5}$ simulated trajectories. Parameters: $t=2.7~\text {s}$ and $t=15.0~\text {s}$, $k_{\text {deg}}^{\text {mRNA}}=1.2~\text {s}^{-1}$, $k_{\text {syn}}^{\text {mRNA}}=1.2~\text {s}^{-1}$, $k_{\text {deg}}^{\text {prot}}=0.3~\text {s}^{-1}$, $k_{\text {transl}}=10.0~\text {s}^{-1}$, deterministic initial conditions with $x_{0}=y_{0}=0$. The thick gray lines depict the expectation value, while the dashed lines stand for the standard deviation. The arrow marks the probability for a zero outcome in the left hand side plot

**Fig. 9:**
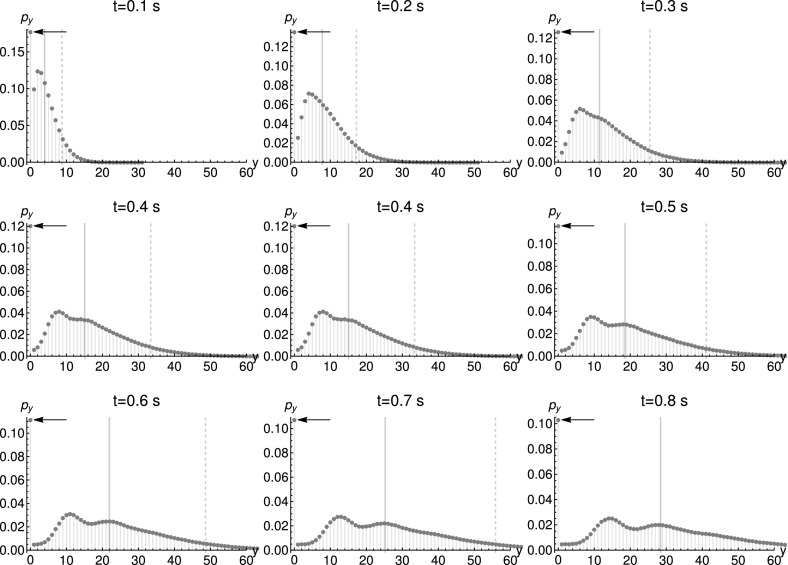
Marginal distribution of proteins $p_{y}:=P(y,t|y_{0}=0,0)$, simulated using Poissonian initial conditions for the mRNA, $\big <x\big >_{0}=2$. Parameters: $k_{\text {deg}}^{\text {mRNA}}=0.2~\text {s}^{-1}$, $k_{\text {syn}}^{\text {mRNA}}=0.4~\text {s}^{-1}$, $k_{\text {deg}}^{\text {prot}}=0.3~\text {s}^{-1}$, $k_{\text {transl}}=20.0~\text {s}^{-1}$. The number of simulated trajectories was $10^{6}$. The thick gray lines depict the expectation value, while the dashed lines stand for the standard deviation. The arrow marks the probability for a zero outcome

The appearance of multimodal distributions is due to the reaction $X\rightarrow X+Y$, studied in Example 2. As mentioned there, the Jacobian’s eigenvalues depend on $s_{Y}$, conditional multimodality is expected. Figures 8 and 9 show that the protein distribution can be multimodal for deterministic and Poissonian initial conditions respectively. Previously, the two-stage transcription–translation model was not shown to be conditionally multimodal (Shahrezaei and Swain 2008). Given small degradation rates, the apparent difference between the shape of Neyman’s Type A distribution (see Figs. 2c and 3), solving the CME for $X\rightarrow X+Y$, and the simulation results is rather small. Furthermore, the previously described “extinction scenario” is visible by the large peak at $y=0$ in Figs. 8 and 9. This highlights the usefulness of basic models for the study of more complex models.

The right panel in Fig. 8 shows that the probability of still having zero proteins after 15 s is much smaller than at 2.6 s. Therefore, the corresponding mode disappears. However, the distribution class is still conditionally multimodal as predicted by Theorem 3. Remember that conditional multimodality is a notion defined by the order of the generalizing distribution $g_{2}(s)$ (see Def. 6), not by the model parameters.

#### Example 6

(Natural decay series including decay particles) In an extension of the classical model by Bateman (1910), we now formulate the reaction network for nuclear decay chains including decay particles $\alpha$, $\beta$ and $\gamma$. The stoichiometry of the model is

$$\begin{aligned} X_{i}\overset{k_{i}}{\rightarrow }X_{i+1}+R_{i,\alpha }\alpha +R_{i,\beta }\beta +R_{i,\gamma }\gamma ~, \end{aligned}$$

where $R_{i,\alpha }$, $R_{i,\beta }$ and $R_{i,\gamma }$ are the numbers of decay particles involved in each reaction.

The generating function PDE corresponding to the Master equation is

41 $$\begin{aligned} \partial _{t}g&=\sum _{i=1}^{n-1}k_{i}(s_{\alpha }^{R_{\alpha ,i}}s_{\beta }^{R_{\beta ,i}}s_{\gamma }^{R_{\gamma ,i}}s_{i+1}-s_{i})\partial _{s_{i}}g. \end{aligned}$$

As before, we solve the PDE (41) using the method of characteristics. The characteristic ODEs corresponding to Eq. (41) are:

42 $$\begin{aligned} \frac{ds_{i}}{dt}&=-k_{i}(s_{\alpha }^{R_{\alpha ,i}}s_{\beta }^{R_{\beta ,i}}s_{\gamma }^{R_{\gamma ,i}}s_{i+1}-s_{i})\qquad (i\in 1,\ldots ,n-1) \end{aligned}$$

43 $$\begin{aligned} \frac{ds_{n}}{dt}&=0 \end{aligned}$$

44 $$\begin{aligned} \frac{ds_{\alpha }}{dt}&=\frac{ds_{\beta }}{dt}=\frac{ds_{\gamma }}{dt}=0\nonumber \\ \frac{dg({\mathbf {s}}(t),t)}{dt}&=0\Rightarrow g({\mathbf {s}}(t),t)=f({\mathbf {s}}^{0}). \end{aligned}$$

These equations represent a hierarchically linear ODE system. It can be interpreted as being composed of two subsystems, just as in Theorem 2. System I consists of all isotopes, while system II represents the $\alpha$, $\beta$ and $\gamma$ particles. System II is a trivial example of a monomolecular reaction network, since no reactions take place within the system, we just have to consider the influx of particles from system I. We note that the Jacobian matrix of the characteristic system is identical to the rate equation’s transposed, as discussed in Sect. 2.3. The ODE system is homogeneous and we have

$$\begin{aligned} {\mathbf {s}}&=e^{{\mathbf {A}}({\mathbf {s_{\alpha ,\beta ,\gamma }}})t}{\mathbf {s}}^{0}\\ \Leftrightarrow {\mathbf {s}}^{0}&=e^{-{\mathbf {A}}({\mathbf {s_{\alpha ,\beta ,\gamma }}})t}{\mathbf {s}}, \end{aligned}$$

where ${\mathbf {A}}({\mathbf {s_{\alpha ,\beta ,\gamma }}})=(\partial _{s_{j}}\frac{ds_{i}}{dt})$ is the Jacobian. We express the generating function for Poissonian initial conditions by this matrix as

$$\begin{aligned} g({\mathbf {s}},t)&=\exp \Big \{\big<{\mathbf {x}}\big>_{0}\cdot ({\mathbf {s}}^{0}-{\mathbf {1}})\Big \}\\&=\exp \Big \{\big<{\mathbf {x}}_{\alpha ,\beta ,\gamma }\big>_{0}\cdot ({\mathbf {s_{\alpha ,\beta ,\gamma }}}-{\mathbf {1}})+\big<{\mathbf {x_{1,\ldots ,n}}}\big>_{0}\cdot (\exp (-{\mathbf {A}}({\mathbf {s_{\alpha ,\beta ,\gamma }}})t){\mathbf {s}}-{\mathbf {1}})\Big \}\\&=\exp \Big \{\big<{\mathbf {x}}_{\alpha ,\beta ,\gamma }\big>_{0}\cdot ({\mathbf {s_{\alpha ,\beta ,\gamma }}}-{\mathbf {1}})+\exp (-{\mathbf {A}}({\mathbf {s_{\alpha ,\beta ,\gamma }}})^{\text {T}}t)\big<{\mathbf {x_{1,\ldots ,n}}}\big>_{0}\cdot {\mathbf {s}}-\big <{\mathbf {x_{1,\ldots ,n}}}\big >_{0}\cdot {\mathbf {1}}\Big \}\ . \end{aligned}$$

In the last line, we have used the identity ${\mathbf {A}}{\mathbf {b}}\cdot {\mathbf {c}}={\mathbf {b}}\cdot {\mathbf {A}}^{\text {T}}{\mathbf {c}}={\mathbf {A}}^{\text {T}}{\mathbf {c}}\cdot {\mathbf {b}}$, which holds for any square matrix ${\mathbf {A}}$ and two vectors ${\mathbf {b}}$, ${\mathbf {c}}$ of the same dimension.^15^ The insight of the equivalence of the characteristic system and the transposed rate equation system, as discussed in Sect. 2.3, greatly simplifies the following computations. In consequence, the solution of the Master equation is not much more difficult than the solution of the rate equations, as provided by Bateman (1910) originally.

The difference to Bateman’s system is that the negative Jacobian matrix ${\mathbf {A}}^{\text {T}}$ depends on the constants $s_{\alpha }$, $s_{\beta }$ and $s_{\gamma }$:

$$\begin{aligned}&-{\mathbf {A}}^{\text {T}}({\mathbf {s_{\alpha ,\beta ,\gamma }}})=\\&~\left( \begin{array}{cccccc} -k_{1} &{} 0 &{} \dots &{} &{} &{} 0\\ k_{1}s_{\alpha }^{R_{\alpha ,1}}s_{\beta }^{R_{\beta ,1}}s_{\gamma }^{R_{\gamma ,1}}\\ 0 &{} \ddots \\ &{} &{} -k_{i} &{} &{} &{} 0\\ &{} &{} k_{i}s_{\alpha }^{R_{\alpha ,i}}s_{\beta }^{R_{\beta ,i}}s_{\gamma }^{R_{\gamma ,i}}\\ &{} &{} &{} \ddots \\ &{} &{} &{} &{} -k_{n-1} &{} 0\\ 0 &{} &{} &{} &{} k_{n-1}s_{\alpha }^{R_{\alpha ,n-1}}s_{\beta }^{R_{\beta ,n-1}}s_{\gamma }^{R_{\gamma ,n-1}} &{} 0 \end{array}\right) . \end{aligned}$$

Since this matrix is triangular, the eigenvalues are the diagonal entries. Because the eigenvalues do not depend on ${\mathbf {s_{\alpha ,\beta ,\gamma }}}$, the marginal distribution of the corresponding particles obeys Proposition 3. Furthermore, Theorem 4 predicts that conditional multimodality depending on the choice of $R_{\alpha ,i}$, $R_{\beta ,i}$ and $R_{\gamma ,i}$. Note that Theorem 4 gives us this information without the exact solution of the system presented here.

We now proceed along the lines of Pressyanov (2002) and solve the rate equation system, transposed to the characteristic system, to obtain $\exp (-{\mathbf {A}}^{\text {T}}({\mathbf {s_{\alpha ,\beta ,\gamma }}})t)\big <{\mathbf {x_{1,\ldots ,n}}}\big >_{0}$. The computations can be found in “Appendix A.4” and yield

$$\begin{aligned}&\Big (\exp \big (-{\mathbf {A}}^{\text {T}}({\mathbf {s_{\alpha ,\beta ,\gamma }}})t\big )\big<{\mathbf {x_{1,\ldots ,n}}}\big>_{0}\Big )_{i}\\&\quad =\big<x_{i}\big>_{0}e^{-k_{i}t}+\sum _{m=1}^{i-1}\big <x_{m}\big >_{0}\Big (\prod _{l=m}^{i-1}k_{l}s_{\alpha }^{R_{\alpha ,l}}s_{\beta }^{R_{\beta ,l}}s_{\gamma }^{R_{\gamma ,l}}\Big )\sum _{l=m}^{i}\frac{e^{-k_{l}t}}{\prod _{j=m,j\not =l}^{i}(k_{j}-k_{l})}~. \end{aligned}$$

In consequence, we use the matrix exponential to express the generating function as

$$\begin{aligned} g({\mathbf {s}},t)=&\exp \Big \{\big<{\mathbf {x}}_{\alpha ,\beta ,\gamma }\big>_{0}\cdot ({\mathbf {s_{\alpha ,\beta ,\gamma }}}-{\mathbf {1}})\\&+\sum _{i=1}^{n}\Bigg [\Bigg (\big<x_{i}\big>_{0}e^{-k_{i}t}\\&\quad +\sum _{m=1}^{i-1}\big<x_{m}\big>_{0}\Big (\prod _{l=m}^{i-1}k_{l}s_{\alpha }^{R_{\alpha ,l}}s_{\beta }^{R_{\beta ,l}}s_{\gamma }^{R_{\gamma ,l}}\Big )\sum _{l=m}^{i}\frac{e^{-k_{l}t}}{\prod _{j=m,j\not =l}^{i}(k_{j}-k_{l})}\Bigg )\Big (s_{i}-\big <x_{i}\big >_{0}\Big )\Bigg ]\Big \}. \end{aligned}$$

Interestingly, the further the isotopes are downstream of the decay chain, the more they contribute to the higher order cumulants^16^ of the $\alpha$, $\beta$ and $\gamma$ particles.^17^ An insight like that is not easily gained by doing stochastic simulations, because the simulation results alone would not directly point to this fact. We also see that the distribution is not simply characterized by mean and covariance.

**Fig. 10:**
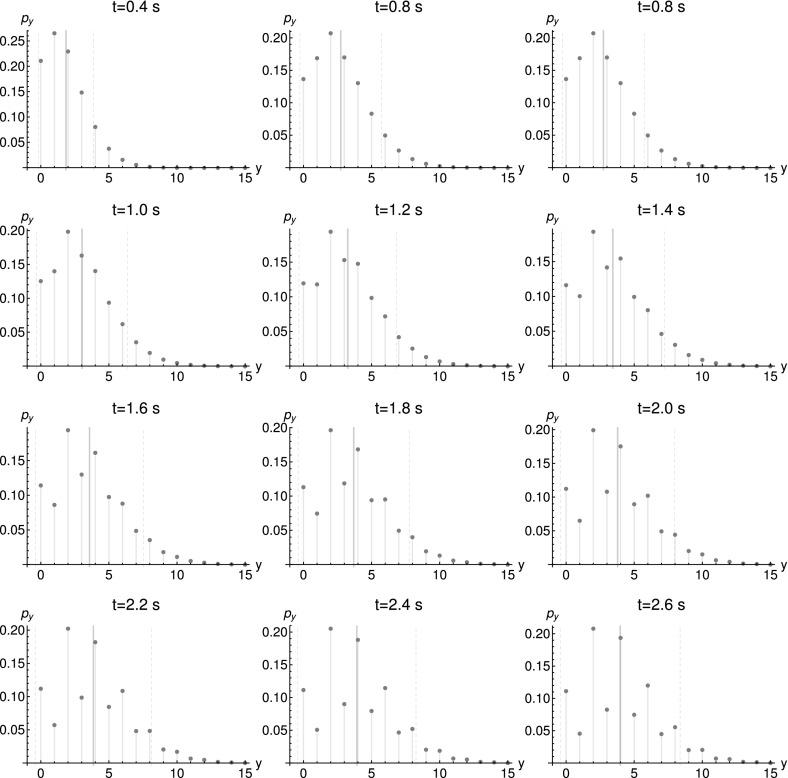
Marginal distribution $p_{y}:=P(y,t|y_{0}=0,0)$ of decay particles *Y*. Parameters: $\big <x_{1}\big >_{0}=2$, $\big <x_{2}\big >_{0}=0.1$, $\big <x_{3}\big >_{0}=0.001$, $k_{1}=3.1~\hbox {s}^{-1}$, $k_{2}=1~\hbox {s}^{-1}$. The thick black line represents the mean while the dashed lines depict the standard deviation

Next, we consider multimodality of the marginal distributions. Since the eigenvalues of ${\mathbf {A}}^{\text {T}}({\mathbf {s_{\alpha ,\beta ,\gamma }}})$ do not depend on ${\mathbf {s_{\alpha ,\beta ,\gamma }}}$, Theorem 4 applies and predicts conditional multimodality for several minimal networks. First, we consider networks corresponding to case 1. We evaluate the solution for the decay chain

45 $$\begin{aligned} X_{1}{\mathop {\rightarrow }\limits ^{k_{1}}}&X_{2}+Y \end{aligned}$$

46 $$\begin{aligned} X_{2}{\mathop {\rightarrow }\limits ^{k_{2}}}&X_{3}+Y \end{aligned}$$

and plot the marginal distributions for *Y* in Fig. 10. The plots, depicting the temporal evolution of the marginal distribution, clearly demonstrate multimodality. Here, (45–46) represent the minimal reaction network Type I from Theorem 4. A minimal reaction network of Type II is obtained by reducing the network to

47 $$\begin{aligned} X_{1}{\mathop {\rightarrow }\limits ^{k_{1}}}X_{2}+2Y. \end{aligned}$$

Finally, by assigning $X_{2}$ to the dependent part of the network, we obtain an even smaller network as $X_{1}{\mathop {\rightarrow }\limits ^{k_{1}}}(X_{2})+2Y$. Now the independent part of the network is open. If we ignore $X_{2}$, this represents case 2 in Theorem 4.

Note that $X_{2}$ can either be assigned to the independent or the dependent part of the reaction network. However, in either case Theorem  4 applies and predicts conditional multimodality.

## Conclusions

In this article, we analyzed the CME for hierarchic first-order reaction networks, based on a general solution method. We showed in Theorem 1 that hierarchic reaction networks are generally treatable in an analytic manner. We derived the analytical solution for the joint probability generating function for Poisson and deterministic initial conditions. Next we analyzed the multimodality of resulting marginal distributions of individual species. The analysis revealed that the marginal distributions of species from the independent part of the network are unimodal (Theorem 2), while the dependent part yields conditionally multimodal distributions (Theorems 3 and 4). Furthermore, we presented several basic models of hierarchic reaction networks, which we consider insightful for the understanding of larger reaction networks. We illustrated this point by showing the similarity between the catalysis basic model and the transcription–translation model. As a proof of principle, we showed that even more complex models such as the nuclear decay chain presented here, are amenable to an exact analytical treatment.

Underlining the prevalence of multimodality, we saw that even trivial networks like the one from Example 1, consisting of the reaction $X\rightarrow X+Y$, are conditionally multimodal given Poissonian initial conditions. We also showed that deterministic initial conditions give rise to multimodal distributions in Examples 2 and 5. For the two-stage transcription–translation model, multimodality was previously not reported (Shahrezaei and Swain 2008).

Since the main Theorems 3 and 4 only require knowledge about the dependence of the eigenvalues of the Jacobian matrix on the variable of interest, it is sufficient to compute the characteristic polynomial of the Jacobian matrix. However, the characteristic polynomial of any square matrix is algorithmically computable and so our Theorems are algorithmically applicable in practice. We furthermore demonstrated how insight about a network may be gained by simply dividing the network into dependent and independent parts (see Example 3), without doing any calculations or simulations. Therefore, we believe that our abstract treatment of hierarchic reaction networks may inspire other researchers to analyze and design networks with respect to their multimodality properties.

## Further developments

As a future project, we suggest the development of a numerical software to determine the parameter regions of multimodality of our basic models. In that study, the collection of basic models maybe extended, since the ones presented here are far from the only ones whose generating function may be written in a compact form. It might also be interesting to study *n*-fold hierarchies instead of the two-level hierarchies presented here.

Furthermore, we note that the Luria–Delbrück model (Zheng 1999), describing the division of wild-type cells *X* into mutants *Y* may be investigated in a similar way we proposed. One of the many variants of the model is defined by

$$\begin{aligned} X&\overset{k_{\text {wt}}}{\rightarrow }2X\\ X&\overset{k_{\text {wt}}^{{\text {mut}}}}{\rightarrow }X+Y\\ X&\overset{d_{\text {wt}}}{\rightarrow }\emptyset \\ Y&\overset{k_{\text {mut}}}{\rightarrow }2Y\\ Y&\overset{d_{\text {mut}}}{\rightarrow }\emptyset . \end{aligned}$$

Even though the reaction equations show the hierarchic character of the model, they do not fit our definition of hierarchic first-order networks. This fact can by seen by the characteristic ODEs:

48 $$\begin{aligned} \frac{ds_{X}}{dt}&=-k_{\text {wt}}(s_{X}^{2}-s_{X})-k_{\text {wt}}^{{\text {mut}}}(s_{X}s_{Y}-s_{X})-d_{\text {wt}}(1-s_{X}) \end{aligned}$$

49 $$\begin{aligned} \frac{ds_{Y}}{dt}&=-k_{\text {mut}}(s_{Y}^{2}-s_{Y})-d_{\text {mut}}(1-s_{Y})\nonumber \\ \frac{dg}{dt}&=0. \end{aligned}$$

The system is not hierarchically linear due to the $s_{X}^{2}$ and $s_{Y}^{2}$ terms, which is why we excluded autocatalytic reactions $X\rightarrow 2X$ from our study. However, (48) and (49) are examples of Riccati differential equations, and may be linearized as such by a coordinate transformation. In this way, an exact solution of the system can be obtained,^18^ even though it involves large expressions involving hypergeometric functions.

### Electronic supplementary material

Below is the link to the electronic supplementary material.

Supplementary material 1 (nb 33 KB)

Supplementary material 2 (nb 37 KB)

Supplementary material 3 (nb 5 KB)

Supplementary material 4 (psc 0 KB)

Supplementary material 5 (nb 198 KB)

Supplementary material 6 (nb 89 KB)

Supplementary material 7 (nb 10 KB)

## Figures and Tables

**Fig. 11:**
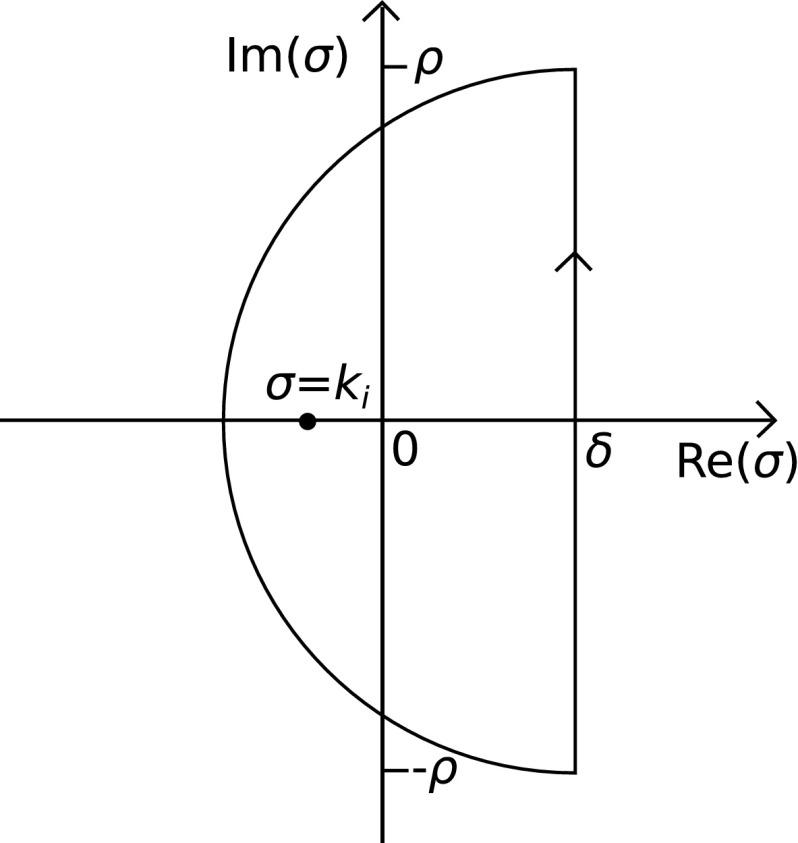
Integration path used for the solution of the Bromwich integral

## Appendix

### A.1 How to obtain distributions from generating functions

Before finally coming to the proof of Proposition 2, we discuss how the probability mass function (PMF), defined as the coefficients of the generating function, can be found. In the first-order case, this is not as easy as in the monomolecular case, where the product Poisson and multinomial distributions are obtained (Jahnke and Huisinga 2007). Even the PMF of the relatively simple Neyman Type A distribution given by $g(s)=e^{\lambda (\exp (\phi (s-1))-1)}$, almost identical to Eq. (38) from Example 1, is defined only in terms of a multinomial expansion (Zhang et al. 2014). In general, the distribution corresponding to the generating function of Eq. (19) is not expressible in terms of elementary functions, except for special cases.

Generally, the coefficients of a generating function $g({\mathbf {s}},t)$ are given by the multivariate Cauchy integral formula

50 $$\begin{aligned} P({\mathbf {x}},t)=\frac{1}{(2\pi i)^{n}}\oint _{{\mathscr {C}}_{1}\times \cdots \times {\mathscr {C}}_{n}}\frac{g({\mathbf {s}},t)}{{\mathbf {s}}^{{\mathbf {x}}+{\mathbf {1}}}}d{\mathbf {s}}. \end{aligned}$$

This identity is due to the definition of the generating function as a power series. Here, ${\mathscr {C}}_{i}$ are discs encircling the origin on the complex (hyper)plane. See Pemantle and Wilson (2013), Appendix A, for the technicalities of the definition of multivariate Cauchy integrals.

Asymptotic joint distributions $|{\mathbf {x}}|\rightarrow \infty$ can be obtained by applying a saddle point integration approach to the Cauchy integral (50). Pemantle and Wilson (2013) present these techniques, developed over the course of the last 20 years, in an excellent textbook. The resulting asymptotic distributions are often in very good numerical agreement with the exact ones, even for low $|{\mathbf {x}}|$, and are a convenient tool to analyze the distributions.

If only a numerical evaluation of the distributions is needed, the Discrete Fourier Transform (DFT) is obtained by defining $s:=e^{-2\pi i\omega }$ with $\omega \in [0,2\pi ]$ and the application of an inverse Fast-Fourier Transform (FFT) leads to a fast computation of the distributions. Recently, Xu and Minin (2015) have made efforts to increase the efficiency of these computations. Numerical integration of (50) is of course also a possibility.

Lastly, it shall be mentioned that there are a number of algorithms based on recurrences (Zheng 1999; Zhang et al. 2014), applicable for one dimensional distributions. The most important of these is the *Lévy*-*Adelson*-*Panjer recursion* for the $\text {DCP}^{N}$, given by

51 $$\begin{aligned} p_{k+1}=\frac{\lambda }{k+1}\sum _{j=0}^{k}(k+1-j)\alpha _{k+1-j}p_{{j}}. \end{aligned}$$

In practice, the sum $\sum _{j=0}^{k}$ often converges after a constant number of terms (i.e. the convergence is linear,^19^) so the computational cost of evaluating *n* probabilities is *O*(*n*). Khatri and Patel (1961) provide recurrences for $\text {KTB}^{N}$ distributions, needed for the deterministic initial condition setting.

The generating function can also be used directly for parameter estimation, without calculating the distribution first. Marcheselli et al. (2008) propose a method for parameter estimation that is based exclusively of generating functions.

### A.2 Proof of Lemma 1

#### Proof

The proof is adapted from Gardiner (2009), Sect. 11.6.4, p. 297. We first multiply with ${\mathbf {s}}^{{\mathbf {x}}}$ and sum over all ${\mathbf {x}}$ to obtain

52 $$\begin{aligned} \partial _{t}g({\mathbf {s}},t)&=\sum _{{\mathbf {x}}={\mathbf {0}}}^{\infty }\sum _{i=1}^{m}{\mathbf {s}}^{{\mathbf {x}}}\left( \alpha _{i}({\mathbf {x}}-{\mathbf {n}}_{i})P({\mathbf {x}}-{\mathbf {n}}_{i},t)-\alpha _{i}({\mathbf {x}})P({\mathbf {x}},t)\right) \nonumber \\&=\sum _{i=1}^{m}\left( \sum _{{\mathbf {x}}'={\mathbf {0}}}^{\infty }{\mathbf {s}}^{{\mathbf {x}}'+{\mathbf {n}}_{i}}\alpha _{i}({\mathbf {x}}')P({\mathbf {x}}',t)-\sum _{{\mathbf {x}}={\mathbf {0}}}^{\infty }{\mathbf {s}}^{{\mathbf {x}}}\alpha _{i}({\mathbf {x}})P({\mathbf {x}},t)\right) . \end{aligned}$$

Using the definition of the stochastic propensities $\alpha _{i}({\mathbf {x}})=k_{i}\prod _{j=1}^{n}\frac{x_{j}!}{(x_{j}-Q_{ji})!}$, we find the identities

$$\begin{aligned} {\mathbf {s}}^{{\mathbf {x}}}\alpha _{i}({\mathbf {x}})&=k_{i}\prod _{j=1}^{n}\frac{s_{j}^{x_{j}}x_{j}!}{(x_{j}-Q_{ji})!}=k_{i}\prod _{j=1}^{n}s_{j}^{x_{j}}(x_{j}(x_{j}-1)(x_{j}-2)\cdots (x_{j}-Q_{ji}+1)\\&=k_{i}\prod _{j=1}^{n}s_{j}^{Q_{ji}}\left( \partial _{s_{j}}^{Q_{ji}}s_{j}^{x_{j}}\right) \end{aligned}$$

and

$$\begin{aligned} {\mathbf {s}}^{{\mathbf {x}}+{\mathbf {n}}_{i}}\alpha _{i}({\mathbf {x}})&=k_{i}\prod _{j=1}^{n}\frac{s_{j}^{x_{j}+n_{ji}}x_{j}!}{(x_{j}-Q_{ji})!}=k_{i}\prod _{j=1}^{n}s_{j}^{R_{ji}}\left( \partial _{s_{j}}^{Q_{ji}}s_{j}^{x_{j}}\right) ~. \end{aligned}$$

Plugging these into Eq. (52), the desired Eq. (4) is obtained. $\square$

### A.3 Proof of solution formula for twofold hierarchy

#### Proof

We start by writing out the PDE for a system of two parts, corresponding to the variables ${\mathbf {s}}_{\text {ind}}:=(s_{1},\ldots ,s_{n_{\text {ind}}})^{\text {T}}$ and ${\mathbf {s}}_{\text {dep}}:=(s_{n_{\text {ind}}+1},\ldots ,s_{n})^{\text {T}}$, as depicted in an exemplary manner in Fig. 1.

The PDE for the generating function, derived from Lemma 1, then reads

$$\begin{aligned} \frac{\partial g({\mathbf {s}},t)}{\partial t}&=\sum _{i=1}^{m}k_{i}\Bigg (c_{i}({\mathbf {s}}_{\text {dep}})\Big (\sum _{l=1}^{n_{\text {ind}}}R_{li}s_{l}+\delta _{\sum _{l=1}^{n_{\text {ind}}}R_{li},0}\Big )-\sum _{l=1}^{n_{\text {ind}}}Q_{li}s_{l}\Bigg )\Big (\sum _{l=1}^{n_{\text {ind}}}Q_{li}\partial _{s_{l}}\Big )g\\&\qquad +\sum _{i=n_{\text {ind}}+1}^{N}\sum _{j=n_{\text {ind}}+1}^{N}\alpha _{ij}\left( s_{j}-s_{i}\right) \partial _{s_{i}}g({\mathbf {s}},t)\\&\qquad +\sum _{i=n_{\text {ind}}+1}^{N}\alpha _{i0}(1-s_{i})\partial _{s_{i}}g({\mathbf {s}},t)+{\mathbf {b}}\cdot ({\mathbf {s}}-{\mathbf {1}})g({\mathbf {s}},t). \end{aligned}$$

$c_{i}({\mathbf {s}}_{\text {dep}}(t',t)):=\prod _{l=n_{\text {ind}}+1}^{n}s_{l}^{R_{li}}(t',t)$ is defined to be the function representing the produced molecules that do not belong to part I. The characteristic ODEs are obtained in the same manner as for monomolecular systems and read for part I as:

$$\begin{aligned} \frac{ds_{j}}{dt}&=-\sum _{i=1}^{m}Q_{ji}k_{i}\left( c_{i}({\mathbf {s}}_{\text {dep}})\Big (\sum _{l=1}^{n_{\text {ind}}}R_{li}s_{l}+\delta _{\sum _{l=1}^{n_{\text {ind}}}R_{li},0}\Big )-Q_{ji}s_{j}\right) \quad (k_{i}\in {\mathbb {R}}^{+}). \end{aligned}$$

Part II is monomolecular and the characteristic equations are:

$$\begin{aligned} \frac{d{\mathbf {s}}_{\text {dep}}}{dt}&=-{\mathbf {A}}^{\text {T}}({\mathbf {s}}_{\text {dep}}-{\mathbf {1}}). \end{aligned}$$

The total derivative of $g({\mathbf {s}}(t),t)$ reads

53 $$\begin{aligned} \frac{dg({\mathbf {s}}(t),t)}{dt}&={\mathbf {b}}\cdot ({\mathbf {s}}-{\mathbf {1}})g({\mathbf {s}},t). \end{aligned}$$

The Jacobian matrix for part I is given by

$$\begin{aligned} J_{jk}&=-\partial _{s_{k}}\sum _{i=1}^{m}Q_{ji}k_{i}\left( c_{i}({\mathbf {s}}_{\text {dep}})\Big (\sum _{l=1}^{n_{\text {ind}}}R_{li}s_{l}(t)+\delta _{\sum _{l=1}^{n_{\text {ind}}}R_{li},0}\Big )-Q_{ji}s_{j}(t)\right) \quad (k\in 1,\ldots n_{\text {ind}})\\&=-\sum _{i=1}^{m}Q_{ji}k_{i}\left( c_{i}({\mathbf {s}}_{\text {dep}})\sum _{l=1}^{n_{\text {ind}}}R_{li}\delta _{kl}-1\right) \\&=\sum _{i=1}^{m}Q_{ji}k_{i}\left( 1-c_{i}({\mathbf {s}}_{\text {dep}})R_{ki}\right) . \end{aligned}$$

If the Jacobian commutes for two different times, i.e. ${\mathbf {J}}(t_{1}){\mathbf {J}}(t_{2})={\mathbf {J}}(t_{2}){\mathbf {J}}(t_{1})$, the homogeneous part of this system is solved by $\exp \Big \{\int _{0}^{t}{\mathbf {J(\mathbf {s}}_{\text {dep}}}(t'){\mathbf {)}}dt'\Big \}$. If it does not commute, $\exp \Big \{\int _{0}^{t}{\mathbf {J(\mathbf {s}}_{\text {dep}}}(t'){\mathbf {)}}dt'\Big \}$ is the first term of the Magnus expansion

$$\begin{aligned} e^{\Omega ({\mathbf {J}}(t))}=\exp \Big \{\int _{0}^{t}{\mathbf {J}}({\mathbf {s}}_{\text {dep}}(t'))dt'+\int _{0}^{t}\int _{0}^{t_{1}}[{\mathbf {J}}({\mathbf {s}}_{\text {dep}}(t_{1})),{\mathbf {J}}({\mathbf {s}}_{\text {dep}}(t_{2}))]dt_{2}dt_{1}+\cdots \Big \}\ . \end{aligned}$$

The nonhomogeneous part is

54 $$\begin{aligned} f_{j}(t):=-\sum _{i=1}^{m}Q_{ji}k_{i}c_{i}({\mathbf {s}}_{\text {dep}}(t)) \end{aligned}$$

for the *l*th component of system I, so the complete solution reads

55 $$\begin{aligned} {\mathbf {s}}_{\text {ind}}(t)&=\exp \Big \{\int _{0}^{t}{\mathbf {J}}({\mathbf {s}}_{\text {dep}}(t'))dt'\Big \}{\mathbf {s}}_{\text {ind}}^{0}+\nonumber \\&\qquad \exp \Big \{\int _{0}^{t}{\mathbf {J}}({\mathbf {s}}_{\text {dep}}(t'))dt'\Big \}\int _{0}^{t}\exp \Big \{-\int _{0}^{t'}{\mathbf {J}}({\mathbf {s}}_{\text {dep}}(t''))dt''\Big \}{\mathbf {f}}({\mathbf {s}}_{\text {dep}}(t'))dt' \end{aligned}$$

56 $$\begin{aligned} {\mathbf {s}}_{\text {dep}}(t)&=\exp (-{\mathbf {A}}^{\text {T}}t)({\mathbf {s}}_{\text {dep}}^{0}-{\mathbf {1}})+{\mathbf {1}}. \end{aligned}$$

From Eq. (53), we know that

$$\begin{aligned} g({\mathbf {s}}(t),t)&=d({\mathbf {s}}^{0})\exp \left( \int _{0}^{t}{\mathbf {b}}\cdot ({\mathbf {s}}(t')-{\mathbf {1}})dt'\right) \end{aligned}$$

where $d({\mathbf {s}}^{0})$ is an arbitrary function to be determined by the initial conditions. The integral over $c_{i}({\mathbf {s}}_{\text {dep}}(t',t))=\prod _{l=n_{\text {ind}}+1}^{n}(s_{l}(t',t))^{R_{li}}$ appears both in the homogeneous and in the inhomogeneous part of the equations for ${\mathbf {s}}_{\text {ind}}(t',t)$:

57 $$\begin{aligned} \int _{0}^{t}\prod _{l=n_{\text {ind}}+1}^{n}s_{l}^{R_{li}}(t',t)dt'&=\int _{0}^{t}\prod _{l=n_{\text {ind}}+1}^{n}\Bigg (\exp \Big \{-{\mathbf {A}}^{\text {T}}t'\Big \}({\mathbf {s}}_{\text {dep}}^{0}-{\mathbf {1}})\cdot \epsilon _{l}+1\Bigg )^{R_{li}}dt'\nonumber \\&=\int _{0}^{t}\prod _{l=n_{\text {ind}}+1}^{n}\Bigg (\exp \Big \{-{\mathbf {A}}^{\text {T}}t'\Big \}\exp ({\mathbf {A}}^{\text {T}}t)({\mathbf {s}}_{\text {dep}}-{\mathbf {1}})\cdot \epsilon _{l}+1\Bigg )^{R_{li}}dt'\nonumber \\&=\int _{0}^{t}\prod _{l=n_{\text {ind}}+1}^{n}\Bigg (\exp \Big \{-{\mathbf {A}}(t'-t)\Big \}\epsilon _{l}\cdot ({\mathbf {s}}_{\text {dep}}-{\mathbf {1}})+1\Bigg )^{R_{li}}dt'. \end{aligned}$$

In the last line, we made use of the identity ${\mathbf {A}}{\mathbf {b}}\cdot {\mathbf {c}}={\mathbf {b}}\cdot {\mathbf {A}}^{\text {T}}{\mathbf {c}}={\mathbf {A}}^{\text {T}}{\mathbf {c}}\cdot {\mathbf {b}}$. We note that the integral can be expressed completely in terms of the fundamental matrix of the second, monomolecular reaction network. Thus, the property that the statistics of monomolecular networks can be fully parametrized by the solution of its traditional reaction rate equations translates to hierarchic first-order networks.

Using Poissonian initial conditions, we find $d({\mathbf {s}}^{0})$:

58 $$\begin{aligned} g({\mathbf {s}}^{0},t=0)=\exp (\left<{\mathbf {x}}\right>_{0}\cdot ({\mathbf {s}}^{0}-{\mathbf {1}}))=d({\mathbf {s}}^{0}). \end{aligned}$$

We solve Eq. (55) for ${\mathbf {s}}_{\text {ind}}^{0}$:

$$\begin{aligned} {\mathbf {s}}_{\text {ind}}^{0}&=\exp \Big \{-\int _{0}^{t}{\mathbf {J}}({\mathbf {s}}_{\text {dep}}(t'))dt'\Big \}{\mathbf {s}}_{\text {ind}}-\int _{0}^{t}\exp \Big \{-\int _{0}^{t'}{\mathbf {J}}({\mathbf {s}}_{\text {dep}}(t''))dt''\Big \}{\mathbf {f(s_{2}}}(t'))dt'. \end{aligned}$$

The last integral is over a double exponential function and a simple exponential, hidden in

$$\begin{aligned} f_{j}({\mathbf {s}}_{\text {dep}},t',t)&:=-\sum _{i=1}^{m}Q_{ji}k_{i}\prod _{l=n_{\text {ind}}+1}^{n}\Bigg (\exp \Big \{-{\mathbf {A}}(t'-t)\Big \}\epsilon _{l}\cdot ({\mathbf {s}}_{\text {dep}}-{\mathbf {1}})+1\Bigg )^{R_{li}} \end{aligned}$$

and is not easily expressed in terms of elementary functions. A series expansion can be found, but is not very simple, which is why we keep the integral as it is. For ${\mathbf {s}}_{\text {dep}}^{0}$, we obtain the same result as in the monomolecular case, namely

$$\begin{aligned} {\mathbf {s}}_{\text {dep}}^{0}&=\exp ({\mathbf {A}}^{\text {T}}t)({\mathbf {s}}_{\text {dep}}-{\mathbf {1}})+{\mathbf {1}}\,. \end{aligned}$$

The complete generating function is

59 $$\begin{aligned} g({\mathbf {s}},t)&=\exp \Big \{\left<{\mathbf {x_{1}}}\right>_{0}\cdot (\underbrace{e^{-\int _{0}^{t}{\mathbf {J}}({\mathbf {s}}_{\text {dep}},t',t)dt'}{\mathbf {s}}_{\text {ind}}-\int _{0}^{t}e^{-\int _{0}^{t'}{\mathbf {J}}({\mathbf {s}}_{\text {dep}},t'',t)dt''}{\mathbf {f}}({\mathbf {s}}_{\text {dep}},t',t)dt'}_{:={\mathbf {s}}_{\text {ind}}(t)}-{\mathbf {1}})\nonumber \\&\qquad +\left<{\mathbf {x_{2}}}\right>_{0}\cdot \exp ({\mathbf {A}}^{{\mathbf {T}}}t)({\mathbf {s}}_{\text {dep}}-{\mathbf {1}})+\int _{0}^{t}{\mathbf {b}}\cdot ({\mathbf {s}}_{\text {ind}}(t''')-{\mathbf {1}})dt'''\Big \} \nonumber \\&=\exp \Big \{(e^{-\int _{0}^{t}{\mathbf {J}}^{\text {T}}({\mathbf {s}}_{\text {dep}},t',t)dt'}\left<{\mathbf {x_{1}}}\right>_{0}\cdot {\mathbf {s}}_{\text {ind}}\nonumber \\&\qquad -\int _{0}^{t}e^{-\int _{0}^{t'}{\mathbf {J}}^{\text {T}}({\mathbf {s}}_{\text {dep}},t'',t)dt''}\left<{\mathbf {x_{1}}}\right>_{0}\cdot {\mathbf {f}}({\mathbf {s}}_{\text {dep}},t',t)dt'-\left<{\mathbf {x_{1}}}\right>_{0})\nonumber \\&\qquad +\exp ({\mathbf {A}}t)\left<{\mathbf {x_{2}}}\right>_{0}\cdot ({\mathbf {s}}_{\text {dep}}-{\mathbf {1}})+\int _{0}^{t}{\mathbf {b}}\cdot ({\mathbf {s}}_{\text {ind}}(t''')-{\mathbf {1}})dt'''\Big \}~. \end{aligned}$$

If the initial condition is deterministic, that is $P({\mathbf {x}},0)=\delta _{{\mathbf {x}},{\mathbf {x}}^{0}}$, where ${\mathbf {x}}^{0}=(x_{1}^{0},\ldots ,x_{n}^{0})^{\text {T}},$ we have

60 $$\begin{aligned} g({\mathbf {s}}(0),0)&=\sum _{{\mathbf {x}}={\mathbf {0}}}^{\infty }\delta _{{\mathbf {x}},{\mathbf {x}}^{0}}({\mathbf {s}}_{0})^{{\mathbf {x}}^{0}}=\prod _{i=1}^{n}(s_{i}^{0})^{x_{i}^{0}}=d({\mathbf {s}}^{0})~. \end{aligned}$$

This yields the result, using $\epsilon _{i}:=\text {col}_{i}{\mathbf {I}}_{n\times n}$ and $\epsilon _{i}^{(1)}:=(\epsilon _{i,1},\ldots ,\epsilon _{i,n_{\text {ind}}})^{\text {T}}$, $\epsilon _{i}^{(2)}:=(\epsilon _{i,n_{\text {ind}}+1},\ldots ,\epsilon _{i,n})^{\text {T}}$:

61 $$\begin{aligned} d({\mathbf {s}}^{0})= & {} \prod _{i=1}^{n}(s_{i}^{0})^{x_{i}^{0}}=\prod _{i=1}^{n}\left( {\mathbf {s}}^{0}\cdot \epsilon _{i}\right) ^{x_{i}^{0}}\nonumber \\= & {} \prod _{i=1}^{n}\Bigg (\big [e^{-\int _{0}^{t}{\mathbf {J}}({\mathbf {s}}_{\text {dep}},t',t)dt'}{\mathbf {s}}_{\text {ind}}-\int _{0}^{t}e^{-\int _{0}^{t'}{\mathbf {J}}({\mathbf {s}}_{\text {dep}},t'',t)dt''}{\mathbf {f}}({\mathbf {s}}_{\text {dep}},t',t)dt'\big ]\cdot \epsilon _{i}^{(1)}\nonumber \\&+\Big (e^{{\mathbf {A}}^{\text {T}}t}({\mathbf {s}}_{\text {dep}}-{\mathbf {1}})+{\mathbf {1}}\Big )\cdot \epsilon _{i}^{(2)}\Bigg )^{x_{i}^{0}}\end{aligned}$$

62 $$\begin{aligned}= & {} \prod _{i=1}^{n}\Bigg (e^{-\int _{0}^{t}{\mathbf {J}}^{\text {T}}({\mathbf {s}}_{\text {dep}}(t'))dt'}\epsilon _{i}^{(1)}\cdot {\mathbf {s}}_{\text {ind}}-\int _{0}^{t}e^{-\int _{0}^{t'}{\mathbf {J}}^{\text {T}}({\mathbf {s}}_{\text {dep}}(t''))dt''}\epsilon _{i}^{(1)}\cdot {\mathbf {f(s_{2}}}(t'))dt'\nonumber \\&+\Big (\exp ({\mathbf {A}}t)\epsilon _{i}^{(2)}\cdot ({\mathbf {s}}_{\text {dep}}-{\mathbf {1}})+1\Big )\Bigg )^{x_{i}^{0}}. \end{aligned}$$

$$\square $$

### A.4 Solution of characteristic ODE system for decay chains

The ODE system, describing the temporal evolution of the concentration of isotope $x_{i}$, is transposed to the characteristic system (43–44) of the PDE (41). More precisely, the system for $x_{i}$ is identical to the original one from Bateman (1910), except for the factors^20^ representing the decay particles, $s_{\alpha }^{R_{\alpha ,i}}s_{\beta }^{R_{\beta ,i}}s_{\gamma }^{R_{\gamma ,i}}$:

63 $$\begin{aligned} \frac{dx_{1}}{dt}= & {} -k_{1}x_{1} \end{aligned}$$

64 $$\begin{aligned} \frac{dx_{2}}{dt}= & {} k_{1}s_{\alpha }^{R_{\alpha ,1}}s_{\beta }^{R_{\beta ,1}}s_{\gamma }^{R_{\gamma ,1}}x_{1}-k_{2}x_{2}\nonumber \\&\vdots \nonumber \\ \frac{dx_{n}}{dt}= & {} k_{n-1}s_{\alpha }^{R_{\alpha ,n-1}}s_{\beta }^{R_{\beta ,n-1}}s_{\gamma }^{R_{\gamma ,n-1}}x_{n-1}-k_{n}x_{n}. \end{aligned}$$

Due to this similarity, we are able to adapt the calculations from Pressyanov (2002) almost one-to-one. We take the Laplace transform^21^ on both sides of Eqs. (63) and (64), thereby introducing the complex variable $\sigma$, setting $x_{i}^{0}=0$ for $i\ge 2$, and obtain

65 $$\begin{aligned} \int _{0}^{\infty }e^{-\sigma t}\frac{dx_{1}}{dt}dt=\int _{0}^{\infty }e^{-\sigma t}dx_{1}= & {} e^{-\sigma t}x_{1}(t)\Big |_{0}^{\infty }+\sigma \int _{0}^{\infty }e^{-\sigma t}x_{1}(t)dt\nonumber \\= & {} -x_{1}^{0}+\sigma \tilde{x}_{1}(\sigma )\nonumber \\= & {} -k_{1}\tilde{x}_{1}(\sigma ), \end{aligned}$$

66 $$\begin{aligned} \int _{0}^{\infty }e^{-\sigma t}dx_{2}= & {} \sigma \tilde{x}_{2}(\sigma )\nonumber \\= & {} k_{1}s_{\alpha }^{R_{\alpha ,1}}s_{\beta }^{R_{\beta ,1}}s_{\gamma }^{R_{\gamma ,1}}\tilde{x}_{1}(\sigma )-k_{2}\tilde{x}_{2}(\sigma )\nonumber \\&\vdots \nonumber \\ \int _{0}^{\infty }e^{-\sigma t}\frac{dx_{n}}{dt}dt= & {} \sigma \tilde{x}_{n}(\sigma )\nonumber \\= & {} k_{n-1}s_{\alpha }^{R_{\alpha ,n-1}}s_{\beta }^{R_{\beta ,n-1}}s_{\gamma }^{R_{\gamma ,n-1}}\tilde{x}_{n-1}(\sigma )-k_{n}\tilde{x}_{n}(\sigma ). \nonumber \\ \end{aligned}$$

In Laplace domain, we solve a linear equation system and invert the Laplace transform $\tilde{{\mathbf {x}}}(\sigma )$ to obtain ${\mathbf {x}}(t)$. The solution of Eqs. (65–66) is given by

67 $$\begin{aligned} \tilde{x}_{1}(\sigma )= & {} \frac{x_{1}^{0}}{k_{1}+\sigma },\nonumber \\ \tilde{x}_{2}(\sigma )= & {} \frac{k_{1}s_{\alpha }^{R_{\alpha ,1}}s_{\beta }^{R_{\beta ,1}}s_{\gamma }^{R_{\gamma ,1}}}{k_{2}+\sigma }\tilde{x}_{2}(\sigma ),\nonumber \\&\vdots \nonumber \\ \tilde{x}_{n}(\sigma )= & {} \frac{k_{n-1}s_{\alpha }^{R_{\alpha ,n-1}}s_{\beta }^{R_{\beta ,n-1}}s_{\gamma }^{R_{\gamma ,n-1}}}{k_{n}+\sigma }\tilde{x}_{n-1}(\sigma ) . \end{aligned}$$

The last Eq. (67) represents the starting point of a linear recurrence, which, if the initial conditions $x_{2},\ldots x_{n}$ are set to zero,^22^ can be solved by

$$\begin{aligned} \tilde{x}_{n}(\sigma )=\frac{\prod _{i=1}^{n-1}k_{i}s_{\alpha }^{R_{\alpha ,i}}s_{\beta }^{R_{\beta ,i}}s_{\gamma }^{R_{\gamma ,i}}}{\prod _{i=1}^{n}(k_{i}+\sigma )}x_{1}^{0}. \end{aligned}$$

Now, we go back from Laplace domain to the time domain, by performing the Bromwich integral

$$\begin{aligned} x_{n}(t)=\frac{1}{2\pi i}\int _{\delta -i\infty }^{\delta +i\infty }e^{\sigma t}\tilde{x}_{n}(\sigma )d\sigma . \end{aligned}$$

Here, $\delta$ defines an integration path parallel to the complex line that is situated right of all singularities of $\tilde{x}_{n}(\sigma )$. We may choose $\delta =0$, since all poles are on the negative part of the real axis. By closing the integration path through the addition of a semicircle (see Fig. 11), we obtain an integral that can be solved by applying the residue theorem, since the contour is closed^23^:

$$\begin{aligned} \frac{1}{2\pi i}\oint _{{\mathscr {C}}}e^{\sigma t}\tilde{x}_{n}(\sigma )d\sigma&=\frac{1}{2\pi i}\int _{-i\infty }^{i\infty }e^{\sigma t}\tilde{x}_{n}(\sigma )d\sigma \\&\quad +\lim _{\rho \rightarrow \infty }\frac{1}{2\pi i}\int _{\pi /2}^{3\pi /2}e^{\rho e^{i\phi }t}\frac{\prod _{i=1}^{n-1}k_{i}s_{\alpha }^{R_{\alpha ,i}}s_{\beta }^{R_{\beta ,i}}s_{\gamma }^{R_{\gamma ,i}}}{\prod _{i=1}^{n}(k_{i}+\rho e^{i\phi })}x_{1}^{0}i\rho e^{i\phi }d\phi . \end{aligned}$$

Here, the contribution of the circular part to the value of the integral along ${\mathscr {C}}$ is zero.^24^ we may calculate $x_{n}(t)$ by the residue formula

$$\begin{aligned} x_{n}(t)=\frac{1}{2\pi i}\oint _{{\mathscr {C}}}e^{\sigma t}\tilde{x}_{n}(\sigma )d\sigma= & {} \sum _{j=1}^{n}\text {Res}_{j}(e^{\sigma t}\tilde{x}_{n}(\sigma ))~, \end{aligned}$$

where the sum is over all poles. In our case, we have *n* simple poles and so the residue theorem yields

$$\begin{aligned} \text {Res}_{j}(e^{\sigma t}\tilde{x}_{n}(\sigma ))= & {} \lim _{\sigma \rightarrow -k_{j}}((\sigma +k_{j})e^{\sigma t}\tilde{x}_{n}(\sigma ))\\= & {} e^{-k_{j}t}\frac{\prod _{i=1}^{n-1}k_{i}s_{\alpha }^{R_{\alpha ,i}}s_{\beta }^{R_{\beta ,i}}s_{\gamma }^{R_{\gamma ,i}}}{\prod _{i=1,i\not =j}^{n}(k_{i}-k_{j})}x_{1}^{0}\ . \end{aligned}$$

Summing over all residues, we get

$$\begin{aligned} x_{n}(t)= & {} x_{1}^{0}\Big (\prod _{i=1}^{n-1}k_{i}s_{\alpha }^{R_{\alpha ,i}}s_{\beta }^{R_{\beta ,i}}s_{\gamma }^{R_{\gamma ,i}}\Big )\sum _{j=1}^{n}\frac{e^{-k_{i}t}}{\prod _{i=1,i\not =j}^{n}(k_{i}-k_{j})}\ . \end{aligned}$$

The only thing left to do is to generalize the initial conditions for $x_{2},\ldots x_{n-1}$. If we set $x_{m}\not =0$, we obtain for example

$$\begin{aligned} x_{i}(t)= & {} x_{1}^{0}\Big (\prod _{l=1}^{i-1}k_{l}s_{\alpha }^{R_{\alpha ,l}}s_{\beta }^{R_{\beta ,l}}s_{\gamma }^{R_{\gamma ,l}}\Big )\sum _{l=1}^{i}\frac{e^{-k_{l}t}}{\prod _{j=1,j\not =l}^{i}(k_{j}-k_{l})}\\&\quad +x_{m}^{0}\Big (\prod _{l=m}^{i-1}k_{l}s_{\alpha }^{R_{\alpha ,l}}s_{\beta }^{R_{\beta ,l}}s_{\gamma }^{R_{\gamma ,l}}\Big )\sum _{l=m}^{i}\frac{e^{-k_{l}t}}{\prod _{j=m,j\not =l}^{l}(k_{j}-k_{l})}. \end{aligned}$$

Applying this idea to all $x_{i}^{0}$, we conclude the solution of the rate equations with

$$\begin{aligned} x_{i}(t)= & {} x_{i}^{0}e^{-k_{i}t}+\\&\quad \sum _{m=1}^{i-1}x_{m}^{0}\Big (\prod _{l=m}^{i-1}k_{l}s_{\alpha }^{R_{\alpha ,l}}s_{\beta }^{R_{\beta ,l}}s_{\gamma }^{R_{\gamma ,l}}\Big )\sum _{l=m}^{i}\frac{e^{-k_{l}t}}{\prod _{j=m,j\not =l}^{i}(k_{j}-k_{l})}\ . \end{aligned}$$

## Footnotes

1 We suppress the dependence on the initial states ${\mathbf {x}}_{0}$ in the following.

2 We suppress the generating function’s dependence on ${\mathbf {x}}_{0}$ and $t_{0}$ in the following.

3 See Evans (2010) for an introduction.

4 The same result may be obtained without the use of generating functions (Jahnke and Huisinga 2007).

5 The temporal dependence of the functions is suppressed as usual. Furthermore, ${\mathbf {x}}_{i}:{\mathbb {R}}_{0}^{+}\rightarrow {\mathbb {C}}^{m_{i}}$, ${\mathbf {f}}_{i}:{\mathbb {C}}^{m_{i+1}}\times {\mathbb {C}}^{m_{i+2}}\times \cdots \times {\mathbb {C}}^{m_{n}}\times {\mathbb {R}}_{0}^{+}\rightarrow {\mathbb {C}}^{m_{i}}$ and ${\mathbf {h}}_{i}:{\mathbb {C}}^{m_{i+1}}\times {\mathbb {C}}^{m_{i+2}}\times \cdots \times {\mathbb {C}}^{m_{n}}\times {\mathbb {R}}_{0}^{+}\rightarrow {\mathbb {C}}^{m_{i}}\times {\mathbb {C}}^{m_{i}}$, where $n,m_{i}\in {\mathbb {N}}$.

6 For a review of the solution of a non-homogeneous system see Brannan (2010).

7 Without this requirement, we obtain an additional factor $e^{\int _{0}^{t}{\mathbf {b}}\cdot ({\mathbf {s}}_{\text {ind}}(t''')-{\mathbf {1}})dt'''}$, where ${\mathbf {s}}_{\text {ind}}(t''')$ is defined in (55). For more compact expressions, this factor was left out without loss of generality.

8 Without this requirement, Magnus series are needed to express the solution. For more compact expressions we restrict ourselves to $[{\mathbf {J}}({\mathbf {s}}_{\text {dep}},t_{1},t),{\mathbf {J}}({\mathbf {s}}_{\text {dep}},t_{2},t)]={\mathbf {0}}$ without loss of generality.

9 The matrix exponential and the following expression, obtained by the Cayley–Hamilton theorem, is reviewed in Rowell (2004).

10 We suppress the time-dependence of ${\mathbf {J}}$ in the following.

11 Here, “$\propto$” means that lower order terms and constant factors in $s_{X}$ are dropped.

12 The time-dependence of ${\mathbf {L}}$ and ${\mathbf {J}}$ is suppressed in the following.

13 Defined by Keilson and Gerber (1971).

14 Note that the coefficients of $e^{\phi (s-1)}$ are defined as the parameters of $\text {DCP}^{\infty }$, not $\phi$.

15 This identity can be seen by writing out the components of the scalar and matrix product:

$$\begin{aligned} \sum _{i=1}^{n}\Big ({\mathbf {A}}{\mathbf {b}}\Big )_{i}c_{i}=\sum _{i=1}^{n}b_{i}\Big ({\mathbf {A}}^{\text {T}}{\mathbf {c}}\Big )_{i}\sum _{i=1}^{n}\sum _{j=1}^{n}A_{ij}b_{j}c{}_{i}=\sum _{i=1}^{n}b_{i}\sum _{j=1}^{n}A_{ij}^{\text {T}}c{}_{j}=\sum _{i=1}^{n}b_{i}\sum _{j=1}^{n}A_{ji}c{}_{j}. \end{aligned}$$

16 The cumulants are defined as the coefficients of the cumulant generating function $\kappa (\xi ):=\log (g(e^{\xi }))$ at $\xi =0$.

17 This can be seen by looking at the number of factors $\prod _{l=m}^{i-1}$.

18 Write the author for a sketch of the calculations.
